# CUL4B orchestrates mesenchymal stem cell commitment by epigenetically repressing KLF4 and C/EBPδ

**DOI:** 10.1038/s41413-023-00263-y

**Published:** 2023-06-02

**Authors:** Ruiqi Yu, Hong Han, Shuxian Chu, Yijun Ding, Shiqi Jin, Yufeng Wang, Wei Jiang, Yuting Liu, Yongxin Zou, Molin Wang, Qiao Liu, Gongping Sun, Baichun Jiang, Yaoqin Gong

**Affiliations:** 1 The Key Laboratory of Experimental Teratology of the Ministry of Education and Department of Genetics, School of Basic Medical Sciences, Cheeloo College of Medicine, Shandong University, Jinan, 250012 China; 2 The Key Laboratory of Liquid‒Solid Structural Evolution and Processing of Materials of Ministry of Education and Institute of Liquid Metal and Casting Technology, School of Materials Science and Engineering, Shandong University, Jinan, 250012 China; 3 The Key Laboratory of Experimental Teratology of the Ministry of Education and Department of Histology and Embryology, School of Basic Medical Sciences, Cheeloo College of Medicine, Shandong University, Jinan, 250012 China

**Keywords:** Bone, Pathogenesis

## Abstract

Dysregulated lineage commitment of mesenchymal stem cells (MSCs) contributes to impaired bone formation and an imbalance between adipogenesis and osteogenesis during skeletal aging and osteoporosis. The intrinsic cellular mechanism that regulates MSC commitment remains unclear. Here, we identified Cullin 4B (CUL4B) as a critical regulator of MSC commitment. CUL4B is expressed in bone marrow MSCs (BMSCs) and downregulated with aging in mice and humans. Conditional knockout of *Cul4b* in MSCs resulted in impaired postnatal skeletal development with low bone mass and reduced bone formation. Moreover, depletion of CUL4B in MSCs aggravated bone loss and marrow adipose accumulation during natural aging or after ovariectomy. In addition, CUL4B deficiency in MSCs reduced bone strength. Mechanistically, CUL4B promoted osteogenesis and inhibited adipogenesis of MSCs by repressing KLF4 and C/EBPδ expression, respectively. The CUL4B complex directly bound to *Klf4* and *Cebpd* and epigenetically repressed their transcription. Collectively, this study reveals CUL4B-mediated epigenetic regulation of the osteogenic or adipogenic commitment of MSCs, which has therapeutic implications in osteoporosis.

## Introduction

Mesenchymal stem cells (MSCs) are a class of adult stem cells that can self-renew and differentiate into multiple cell lineages, such as osteoblasts, chondrocytes and adipocytes.^1,2^ The differentiation of MSCs into adipocytes and osteoblasts is inversely related and must be delicately balanced.^3^ Dysregulation of the osteo-adipogenic balance results in various pathophysiologic processes, including age-related osteoporosis.^4,5^ Under aging or other pathological conditions, such as hormone disorders, bone marrow MSCs (BMSCs) favor adipogenic to osteogenic differentiation, resulting in excessive accumulation of marrow adipose tissue (MAT) and decreased osteoblasts, leading to osteoporosis.^6^

Adipogenesis is directed by peroxisome proliferation-activated receptor γ (PPARγ) and CCAAT/enhancer binding protein α (C/EBPα), which are induced by C/EBPδ.^7,8^ In contrast, Runt-related transcription factor 2 (RUNX2) and OSTERIX are considered master regulators of osteogenic differentiation.^9,10^ Kruppel-like factor 4 (KLF4) can directly interact with RUNX2 to suppress its transcriptional activity and attenuate osteoblast formation.^11^ This molecule can also promote early adipogenesis by activating C/EBPβ transcription.^12^ Although identification of these key regulators improves our understanding of osteogenesis and adipogenesis, how the osteo-adipogenic balance of MSC commitment is regulated remains unclear.

Cullin 4B (CUL4B) is a scaffold protein of CUL4B-RING E3 ligase (CRL4B) complexes involved in diverse physiological and developmental processes. CRL4B can catalyze polyubiquitination of substrate proteins for proteasomal degradation as well as monoubiquitination of H2AK119 for transcriptional repression.^13^ Mutations in the human *CUL4B* gene are a common cause of X-linked mental retardation syndrome.^14,15^ Patients with *CUL4B* mutations manifest mental retardation, short stature, brachydactyly, and central obesity, suggesting a potential role of CUL4B in the regulation of adipogenesis and osteogenesis and their balance. Using an adipocyte-specific *Cul4b* knockout mouse model, we previously showed that the CRL4B complex promotes the degradation of PPARγ to suppress adipogenesis.^16^ Here, we investigated the role of CUL4B in orchestrating osteogenic or adipogenic differentiation of MSCs using a conditional knockout mouse model. Loss of CUL4B in MSCs impaired postnatal skeletal development and aggravated the effect of aging or ovariectomy on MAT accumulation and bone loss. Mechanistically, we identified KLF4 and C/EBPδ, two critical regulators of osteogenic and adipogenic differentiation, as downstream effectors of CUL4B in regulating MSC differentiation. Our work reveals a new epigenetic mechanism underlying the regulation of the osteo-adipogenic balance of MSC differentiation and suggests CUL4B as a potential therapeutic target in osteoporosis treatment.

## Results

### CUL4B expression in BMSCs declines during aging

**Fig. 1:**
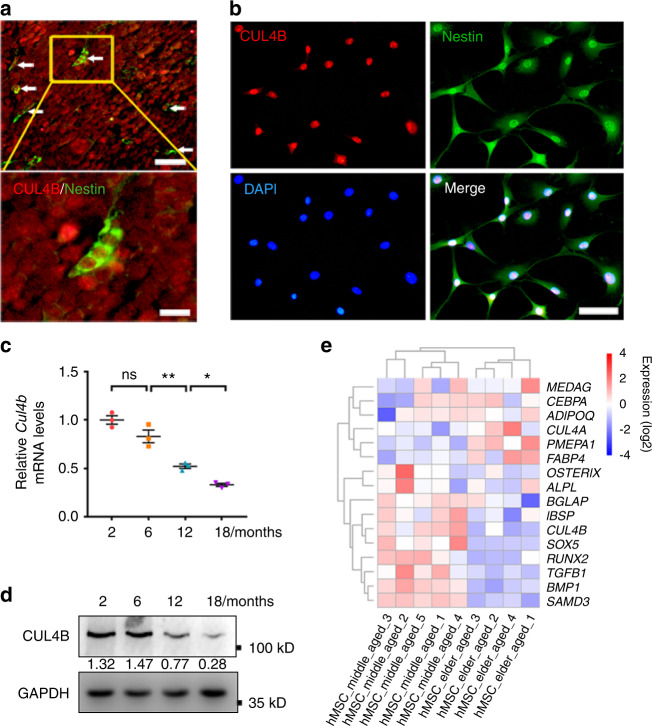
CUL4B expression in BMSCs declines during aging. **a** Representative images of immunofluorescence (IF) analysis showing the overlap of CUL4B (red) and Nestin (green) in fibroblast-like cells (white arrows) in the bone marrow of 1-month-old mice. The scale bars are 20 μm (upper panel) and 6 μm (lower panel). **b** IF showed colocalization of CUL4B (red) and Nestin (green) in cultures of BMSCs isolated from bone marrow of 1-month-old mice. Scale bar, 20 μm. **c** Relative mRNA levels of *Cul4b* from BMSCs of 2-, 6-, 12- and 18-month-old mice (*n* = 3) were determined by qPCR. **d** Protein levels of CUL4B from BMSCs of 2-, 6-, 12- and 18-month-old mice were determined by Western blotting. **e** Heatmap of genes in BMSCs from middle-aged and elderly people analyzed from microarray data of the Gene Expression Omnibus (GEO) Database (GSE35955). Error bars represent standard errors. *P < 0.05; **P < 0.01; ns, no significance

We first analyzed the expression of CUL4B in BMSCs. Since nestin is a marker for a subpopulation of BMSCs,^17^ we examined the colocalization of CUL4B and nestin by immunofluorescence (IF) staining. As shown in Fig. 1a, b, CUL4B was detected in nestin-positive cells in both bone marrow (Fig. 1a) and isolated BMSCs (Fig. 1b). Interestingly, both the mRNA and protein levels of CUL4B declined progressively in mouse BMSCs from 2 months to 18 months (Fig. 1c, d). Analysis of previously published microarray data (GSE35955)^18^ also revealed that *CUL4B* levels in BMSCs from aged people (79–89 years) were lower than those in BMSCs from middle-aged people (42–67 years; Fig. 1e). These results suggest that CUL4B expression in BMSCs declines during aging in both mice and humans, indicating the important roles of CUL4B in the commitment of BMSCs along with aging.

### Depletion of CUL4B in MSCs impairs postnatal skeletal development

**Fig. 2:**
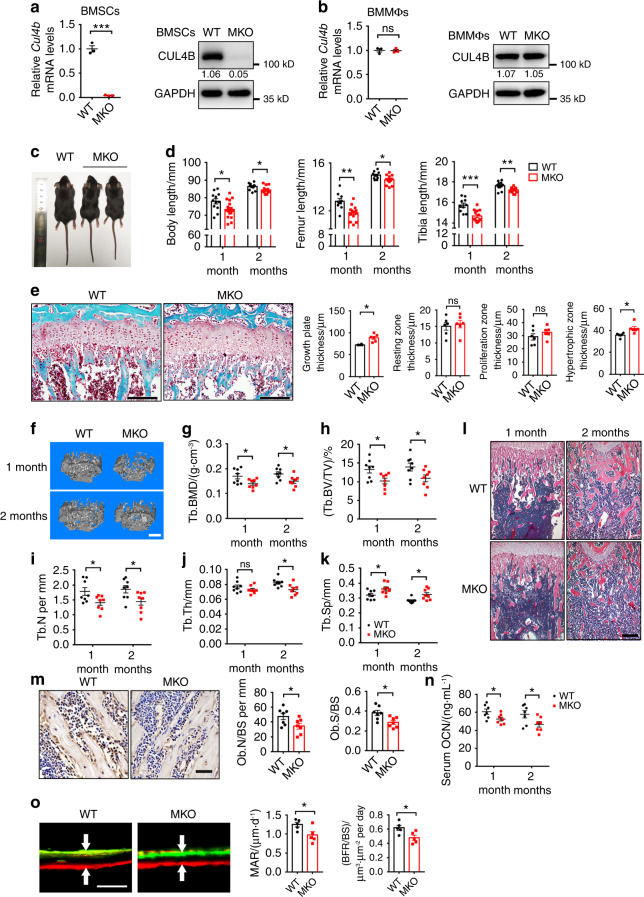
Depletion of CUL4B in MSCs impaired postnatal skeletal development. **a** The expression of CUL4B in cultured BMSCs of WT and MKO mice (*n* = 3) was determined by qPCR and Western blotting. **b** The expression of CUL4B in bone marrow monocytes and/or macrophages (BMMΦs) of WT and MKO mice (*n* = 3) was determined by qPCR and Western blotting. **c** Gross appearance of WT and MKO mice at 1 month. **d** Body length, femur length and tibia length of WT and MKO mice at the ages of 1 (*n* = 12;18) and 2 months (*n* = 11;12). **e** Safranin O staining of paraffin sections from the tibia of 2-month-old WT and MKO mice. Representative images are shown with quantifications of the thickness of the growth plate and its resting zone, proliferation zone and hypertrophic zone (*n* = 6). **f** Representative micro-CT three-dimensional reconstruction images of trabecular bone of femurs from 1- and 2-month-old WT and MKO mice. Scale bar, 0.3 mm. **g**–**k** Quantitative measurements of the trabecular bone mineral density (BMD) (**g**), bone volume/total volume (BV/TV) (**h**), trabecular number (Tb.N) (**i**), trabecular thickness (Tb.Th) (**j**) and trabecular separation (Tb.Sp) (**k**) of femurs from 1- (*n* = 8;8) and 2-month-old (*n* = 8;8) WT and MKO mice by micro-CT. **l** Representative H&E staining of 1- and 2-month-old mouse femurs. Scale bar, 200 μm. **m** Representative IHC images of OCN, osteoblast number (Ob. N/BS) and osteoblast surface (Ob. S/BS) in 1-month-old WT and MKO mice (*n* = 7). Scale bar, 40 μm. **n** ELISAs of serum OCN in 1- (*n* = 8) and 2-month-old (*n* = 7) WT and MKO mice. **o** Alizarin red and calcein double fluorochrome labeling of trabecular bone in femur metaphysis from 2-month-old WT and MKO mice. Representative images are shown with quantifications of the mineral apposition rate (MAR) and bone formation rate (BFR) (*n* = 5). Error bars represent standard errors. *P < 0.05; **P < 0.01; ***P < 0.001; ns, no significance

*Prx1*-*Cre* is expressed throughout the early limb bud mesenchyme and can be used to delete genes in the mesenchymal lineage.^19^ To explore the role of CUL4B in BMSCs, we crossed *Prx1*-*Cre* mice with *Cul4b* floxed mice to generate mice with *Cul4b* conditionally knocked out in MSCs (*Prx1*-*Cre*^*+/−*^;*Cul4b*^*flox/Y*^, referred to as MKO hereafter; Fig. S1a). *Prx1*-*Cre*^−/−^;*Cul4b*^*flox/Y*^ littermates were used as controls (WT). Depletion of CUL4B in CD29^+^/CD44^+^/CD105^+^/Sca-1^+^/CD45^−^/CD11b^−^/CD14^−^/CD34^−^ BMSCs^3,20^ in MKO mice was confirmed by qPCR and Western blotting (Fig. 2a and Fig. S1b), whereas the expression of CUL4B in monocytes and/or macrophages was not changed (Fig. 2b).

The MKO mice were indistinguishable from the littermate controls in size at birth (Fig. S2a). Whole-mount skeletal staining showed that the MKO and control newborns exhibited comparable skeletal patterning with both cartilaginous and calcified skeletal elements (Fig. S2b, c). The body length of the MKO mice appeared slightly shorter than that of the control mice at the age of 1 month (Fig. 2c). Further analysis showed that the body length as well as the femur and tibia length of the 1- and 2-month-old MKO mice were significantly shorter than those of the age-matched control mice (Fig. 2d), which recaptures the short stature phenotype in human patients with *CUL4B* mutation. Safranin O staining showed a striking expansion of the tibial growth plate in the MKO mice compared to that in the controls at the age of 2 months, and this difference was mainly due to the increased hypertrophic zone of the growth plate (Fig. 2e), suggesting that CUL4B may regulate endochondral bone formation.

Microcomputed tomography (micro-CT) analysis of femurs from 1- and 2-month-old mice revealed that the trabecular bone mineral density (BMD) was reduced in the MKO mice compared to the control mice (17.47% decreased at 1 month, 16.39% decreased at 2 months) (Fig. 2f, g). The trabecular bone volume/tissue volume ratio (BV/TV) of the MKO mice was also reduced (23.22% decreased at 1 month, 21.78% decreased at 2 months) (Fig. 2h). In addition, the MKO mice showed decreased trabecular number (Tb.N) (Fig. 2i) and trabecular thickness (Tb.Th) (Fig. 2j) and increased trabecular separation (Tb. Sp) (Fig. 2k). Although depletion of CUL4B in MSCs did not affect the cortical bone thickness, cross-sectional area or porosity, the BMD of cortical bone in the femur diaphysis of the MKO mice was lower than that in the control mice (Fig. S3). Consistent with the micro-CT results, H&E staining revealed reduced trabecular bones in the femurs and tibias of the 1- and 2-month-old MKO mice compared to the age-matched control mice (Fig. 2l, Fig. S4a).

Histomorphometric analysis of staining for osteocalcin (OCN), a marker for mature osteoblasts, revealed fewer osteoblasts (Ob.N) and a smaller osteoblast surface (Ob.S) in the 1-month-old MKO mice than the control mice (Fig. 2m). The serum OCN concentration was also lower in the 1- and 2-month-old MKO mice than in the control mice (Fig. 2n). Double fluorochrome labeling experiments showed that the mineral apposition rate (MAR) and bone formation rate (BFR) of trabecular bone in the femur metaphysis of the MKO mice were significantly lower than those in the control mice (Fig. 2o). These results demonstrate that depletion of *Cul4b* in MSCs reduces bone formation. Intriguingly, osteoclast number (Oc.N) and osteoclast surface (Oc.S) were higher in the MKO mice than in the control mice (Fig. S5a, b). The concentration of serum Trap5b, a marker for bone resorption, was also higher in the 2-month-old MKO mice than in the control mice (Fig. S5c).

### Depletion of CUL4B in MSCs exacerbates age-related bone loss and MAT accumulation

**Fig. 3:**
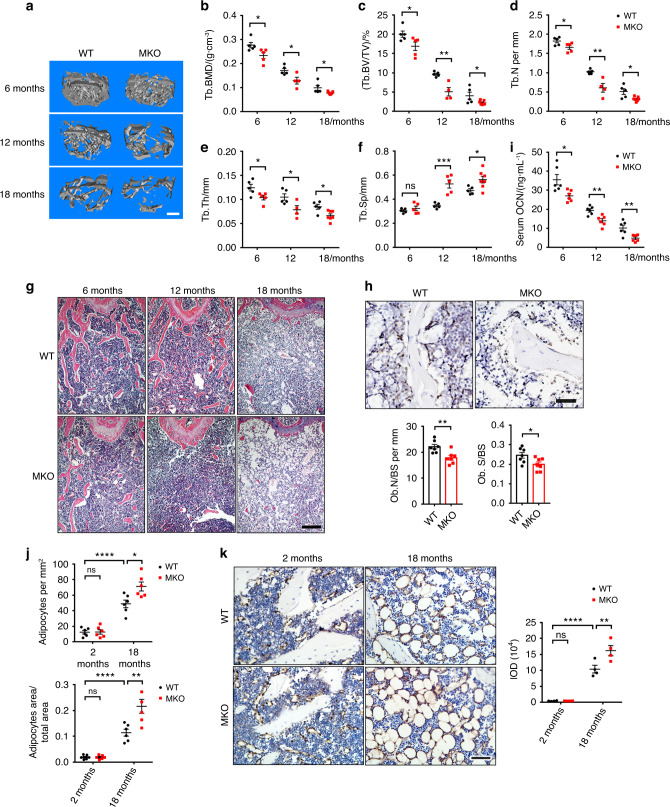
Depletion of CUL4B in MSCs exacerbated age-related bone loss and MAT accumulation. **a** Representative micro-CT three-dimensional reconstruction images of trabecular bone of femurs from 6-, 12- and 18-month-old WT and MKO mice. Scale bar, 0.3 mm. **b**–**f** Quantitative measurements of the trabecular bone mineral density (BMD) (**b**), bone volume/total volume (BV/TV) (**c**), trabecular number (Tb.N) (**d**), trabecular thickness (Tb.Th) (**e**) and trabecular separation (Tb.Sp) (**f**) of femurs from 6- (*n* = 5;5), 12- (*n* = 5;4) and 18-month-old (*n* = 5;7) WT and MKO mice by micro-CT. **g** Representative H&E staining of 6-, 12- and 18-month-old mouse femurs. Scale bar, 200 μm. **h** Representative IHC images of OCN, osteoblast number (Ob.N/BS) and osteoblast surface (Ob.S/BS) in 12-month-old WT and MKO mice (*n* = 7). Scale bar, 40 μm. **i** ELISAs of serum OCN in 6- (*n* = 6), 12- (*n* = 6) and 18-month-old (*n* = 6) WT and MKO mice. **j** Adipocyte number per tissue area and area of adipocytes per tissue area measured based on H&E images of 2- (*n* = 6) and 18-month-old (*n* = 6) WT and MKO mouse femurs. **k** Representative IHC of FABP4 and IOD quantification of 2- (*n* = 4) and 18-month-old (*n* = 4) WT and MKO mice. Scale bar, 40 μm. Error bars represent standard errors. *P < 0.05; **P < 0.01; ***P < 0.001; ****P < 0.000 1; ns, no significance

We next evaluated whether depletion of CUL4B in MSCs affects aging-related bone loss. Micro-CT analysis revealed that the trabecular bone mass of femurs in both control and MKO mice was gradually reduced from 6 to 18 months (Fig. 3a). While the BMD was decreased in the MKO mice compared to that in the control mice at all ages, the difference between the two genotypes was greater in older mice (15.60% at 6 months, 24.81% at 12 months, 21.66% at 18 months) (Fig. 3b). Similarly, the BV/TV was also reduced in the MKO mice compared to the control mice, with a greater difference at older ages (15.74% at 6 months, 47.30% at 12 months, 44.77% at 18 months) (Fig. 3c). The reductions in the average BMD and BV/TV from 6 to 12 months old were greater in the MKO mice than in the control mice (38.07% in control vs. 44.83% in MKO for BMD and 51.98% in control vs. 69.96% in MKO for BV/TV; Fig. 3b, c). Moreover, the BMD and BV/TV values of 12-month-old MKO mice were similar to those of 18-month-old control mice (Fig. 3b, c). The MKO mice at all ages had decreased Tb.N (Fig. 3d) and Tb.Th (Fig. 3e) and increased Tb.Sp (Fig. 3f) compared to the age-matched control mice. The MKO mice also exhibited more trabecular bone loss (Fig. 3g, Fig. S4a) and less mineralized tissues in the femurs than the control mice (Fig. S4b).

A recent study reported that knockout of genes with *Prx1*-*Cre* can affect vertebral bone in adult and aged mice,^21^ so we also examined the vertebrae by micro-CT analysis and found reduced bone mass in the vertebrae of MKO mice at the ages of 6 and 12 months (Fig. S6). Although the thickness and cross-sectional area of the cortical bone of femurs were similar between aged MKO and control mice (Fig. S7a–c), the cortical BMD was decreased and the cortical porosity was increased in the femur diaphysis of the aged MKO mice (Fig. S7d, e).

IHC analysis of OCN showed that at 18 months old, MKO mice had lower osteoblast numbers and osteoblast surfaces than control mice (Fig. 3h). MKO mice also exhibited reduced concentrations of serum OCN compared to age-matched control mice from 6 to 18 months old (Fig. 3i). In contrast, osteoclasts and serum Trap5b levels were increased in the aged MKO mice (Fig. S5).

In addition, we observed marked changes in MAT accumulation via H&E staining (Fig. 3g). Compared to 2-month-old mice, 18-month-old mice exhibited significantly increased adipocyte number and adipocyte area. Importantly, this adipocyte accumulation was dramatically exacerbated in MKO mice (Fig. 3j). IHC for FABP4, an adipocyte marker, confirmed the exacerbation of adipocyte accumulation in MKO mice (Fig. 3k). Taken together, these results indicate that depletion of CUL4B in MSCs exacerbates age-related bone loss and MAT accumulation.

### Depletion of CUL4B in MSCs aggravates estrogen deficiency-induced bone loss and MAT accumulation

**Fig. 4:**
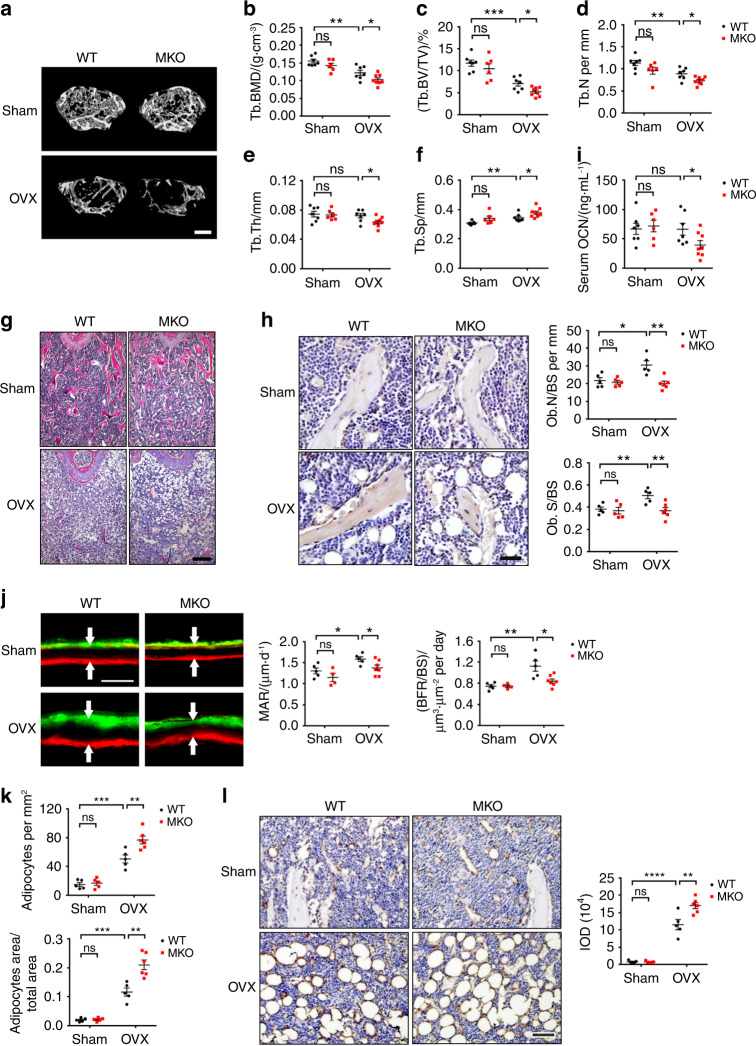
CUL4B exacerbated bone loss and MAT accumulation induced by OVX. **a** Representative micro-CT three-dimensional reconstruction images of trabecular bone of femurs from WT and MKO mice after sham or OVX surgery. Scale bar, 0.3 mm. **b**–**f** Quantitative measurements of the trabecular bone mineral density (BMD) (**b**), bone volume/total volume (BV/TV) (**c**), trabecular number (Tb.N) (**d**), trabecular thickness (Tb.Th) (**e**) and trabecular separation (Tb.Sp) (**f**) of femurs from WT and MKO mice after sham (*n* = 7;6) or OVX surgery (*n* = 7;8) by micro-CT. **g** Representative H&E staining of femurs from WT and MKO mice after sham or OVX surgery. Scale bar, 200 μm. (**h**) Representative IHC images of OCN, osteoblast number (Ob.N/BS) and osteoblast surface (Ob.S/BS) in WT and MKO mice after sham (*n* = 5;5) or OVX surgery (*n* = 5;6). Scale bar, 40 μm. **i** ELISAs of serum OCN in WT and MKO mice after sham (*n* = 7;6) or OVX surgery (*n* = 7;8). **j** Alizarin red and calcein double fluorochrome labeling of trabecular bone in femur metaphysis from WT and MKO mice after sham (*n* = 5;4) or OVX surgery (*n* = 5;7). Scale bar, 20 μm. Representative images are shown with quantifications of the mineral apposition rate (MAR) and bone formation rate (BFR). **k** Adipocyte number per tissue area and area of adipocytes per tissue area measured based on H&E images of femurs from WT and MKO mice after sham (*n* = 5;5) or OVX surgery (n = 5;6). **l** Representative IHC of FABP4 and IOD quantification of WT and MKO mice after sham (*n* = 5;5) or OVX surgery (*n* = 5;6). Scale bar, 40 μm. Error bars represent standard errors. *P < 0.05; **P < 0.01; ***P < 0.001; ****P < 0.000 1; ns, no significance

Mouse ovariectomy (OVX) is a classic model to induce postmenopausal osteoporosis. We thus performed a sham or OVX operation on control and MKO mice to investigate whether conditional deletion of CUL4B in MSCs affected osteoporotic bone loss and MAT accumulation in mice following OVX. Micro-CT analysis of femurs showed noticeable trabecular bone loss in the control mice at 1 month after OVX compared to the sham controls. As expected, bone loss was markedly aggravated in the MKO mice after OVX (Fig. 4a). While there were no significant differences in the two groups after sham operation, the BMD was reduced by 15.94% and BV/TV was reduced by 26.14% in the MKO mice compared to those in the control mice after OVX (Fig. 4b, c). The OVX-induced reductions in BMD and BV/TV were greater in the MKO mice than in the control mice (21.27% in control vs. 28.00% in MKO for BMD and 39.65% in control vs. 50.09% in MKO for BV/TV) (Fig. 4b, c). Tb.N (Fig. 4d) and Tb.Th (Fig. 4e) were decreased, and Tb.Sp (Fig. 4f) was increased in the MKO mice after OVX. The aggravated trabecular bone loss in the MKO mice after OVX was further confirmed by H&E staining of femurs (Fig. 4g). IHC analysis of OCN revealed decreased Ob.N and Ob.S in the MKO mice compared to the control mice after OVX (Fig. 4h). The concentration of serum OCN was also lower in the MKO mice than in the control mice after OVX (Fig. 4i). Similarly, BFR and MAR were elevated in the control mice after OVX to compensate for the accelerated bone resorption, whereas BFR and MAR were significantly decreased in the MKO mice compared to the control mice after OVX (Fig. 4j). Moreover, the MKO mice displayed more adipocytes and larger adipocyte areas than the control mice after OVX (Fig. 4k). IHC for FABP4 confirmed the exacerbation of adipocyte accumulation in the MKO mice after OVX (Fig. 4l). These data suggest that depletion of CUL4B in MSCs aggravates estrogen deficiency-induced bone loss and MAT accumulation.

### Loss of CUL4B in MSCs reduces bone strength

**Fig. 5:**
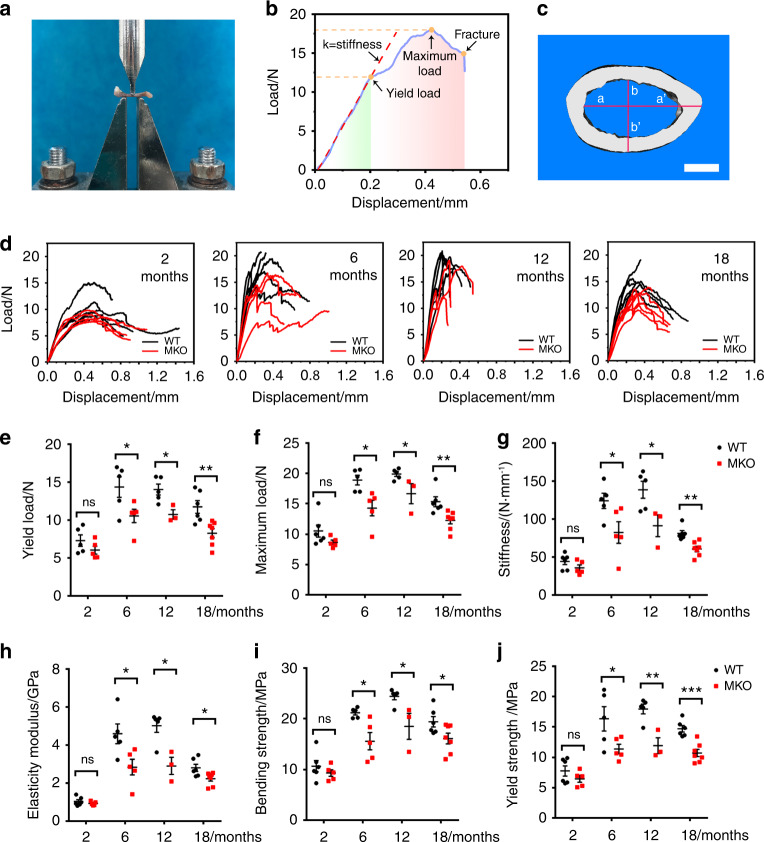
Depletion of CUL4B in MSCs reduced bone strength. **a** Femur loaded in the three-point bending test. **b** Load‒displacement curve from a femur showing yield load, maximum load, fracture point and stiffness. **c** Three-dimensional reconstructed cross section of femur for the calculation of moment of inertia, yield strength, and bending strength using ImageJ. Scale bar, 0.5 mm. **d** Load‒displacement curves from 2-, 6-, 12- and 18-month-old WT and MKO femurs. **e**–**j** The biomechanical parameters of 2- (*n* = 6;5), 6- (*n* = 5), 12- (*n* = 5;3) and 18-month-old (*n* = 6;7) WT and MKO femurs: yield load (**e**), maximum load (**f**), stiffness (**g**), elasticity modulus (**h**), bending strength (**i**) and yield strength (**j**). Error bars represent standard errors. *P < 0.05; **P < 0.01; ***P < 0.001; ns, no significance

To determine whether depletion of CUL4B from MSCs affected bone strength, we quantified the biomechanical properties of the femurs by a three-point bending test (Fig. 5a–c). As shown in Fig. 5d, load‒displacement curves were analyzed for mechanical properties, including stiffness, maximum load, displacement at maximum load, load at yield, displacement at yield, load at fracture, and displacement at fracture, and revealed differences between the control and MKO mice. All biomechanical parameters (including yield load, maximum load, stiffness, elasticity modulus, bending strength, and yield strength) of the femurs from the skeletally mature mice (e.g., 6-, 12- and 18-month-old) were markedly higher than those of 2-month-old mice, whereas these parameters decreased at 18 months of age compared to those at 12 months. Importantly, once the mice reached skeletal maturity, all biomechanical parameters of the MKO mice were markedly lower than those of the control mice (Fig. 5e–j). Taken together, these results show that MKO mice have lower bone strength than age-matched control mice, suggesting that MKO mice have an increased risk of fracture.

### CUL4B regulates osteogenic and adipogenic differentiation of BMSCs in vitro

**Fig. 6:**
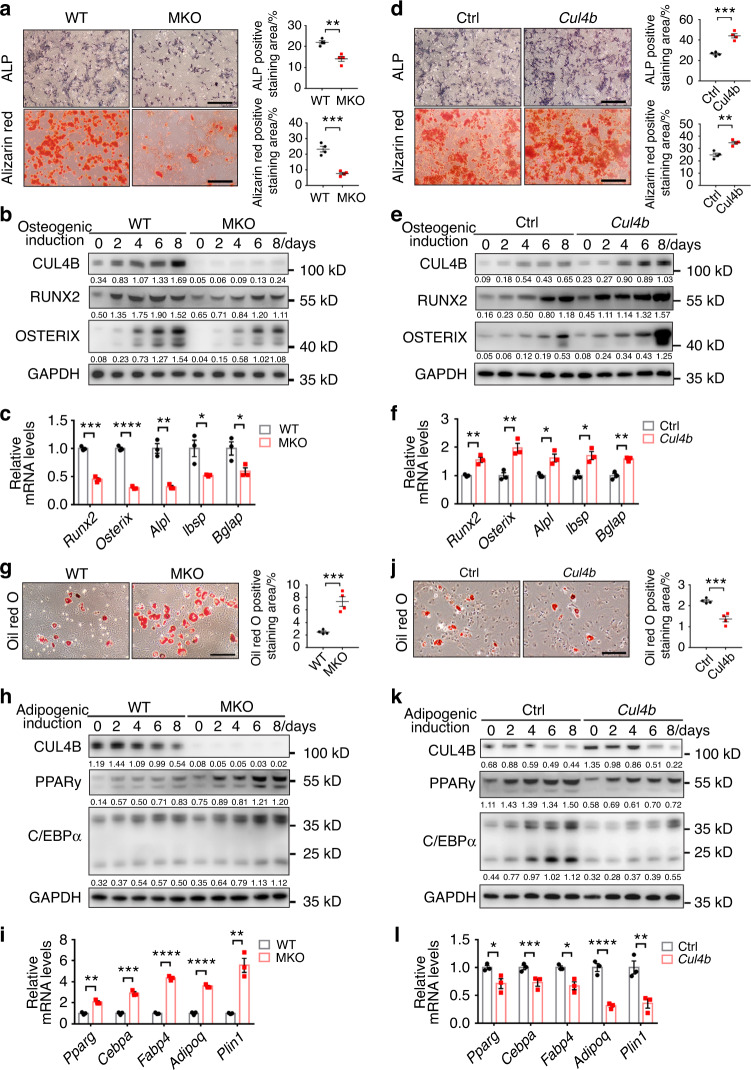
CUL4B regulates osteogenic and adipogenic differentiation of BMSCs in vitro. **a** Osteogenic differentiation of BMSCs from MKO and WT mice (*n* = 4) was determined by ALP activity (upper panels) and alizarin red staining (lower panels). Scale bar, 200 μm. **b** The protein levels of the key osteogenic regulators RUNX2 and OSTERIX in MKO and WT BMSCs during osteogenic differentiation were detected by Western blotting. **c** The expression of key osteogenic marker genes, including *Runx2*, *Osterix*, *Alpl*, *Ibsp* and *Bglap*, in MKO and WT BMSCs (*n* = 4) after osteogenic induction was detected by qPCR. **d** Osteogenic differentiation of CUL4B-overexpressing and control BMSCs (*n* = 4) was determined by ALP activity (upper panels) and alizarin red staining (lower panels). Scale bar, 200 μm. **e** The protein levels of the key osteogenic regulators RUNX2 and OSTERIX in CUL4B-overexpressing and control BMSCs during osteogenic differentiation were detected by Western blotting. **f** The expression of key osteogenic marker genes, including *Runx2*, *Osterix*, *Alpl*, *Ibsp* and *Bglap*, in CUL4B-overexpressing and control BMSCs (*n* = 4) after osteogenic induction was detected by qPCR. **g** Adipogenic differentiation of BMSCs from MKO and WT mice (*n* = 4) was determined by oil red O staining. Scale bar, 60 μm. **h** The protein levels of the key adipogenic regulators PPARγ and C/EBPα in MKO and WT BMSCs during adipogenic differentiation were detected by Western blotting. **i** The expression of key adipogenic marker genes, including *Pparg*, *Cebpa*, *Fabp4*, *Adipoq* and *Plin1*, in MKO and WT BMSCs (*n* = 4) after adipogenic induction was detected by qPCR. **j** Adipogenic differentiation of CUL4B-overexpressing and control BMSCs (*n* = 4) was determined by oil red O staining. Scale bar, 60 μm. **k** The protein levels of the key adipogenic regulators PPARγ and C/EBPα in CUL4B-overexpressing and control BMSCs during adipogenic differentiation were detected by Western blotting. **l** The expression of key adipogenic marker genes, including *Pparg*, *Cebpa*, *Fabp4*, *Adipoq* and *Plin1*, in CUL4B-overexpressing and control BMSCs (*n* = 4) after adipogenic induction was detected by qPCR. Error bars represent standard errors. *P < 0.05; **P < 0.01; ***P < 0.001; ****P < 0.000 1

To further determine the role of CUL4B in BMSC fate decisions, we investigated the effect of CUL4B on the osteo-adipogenic differentiation of BMSCs in vitro. Under osteogenic induction, BMSCs from MKO mice showed less alkaline phosphatase (ALP) activity than those from control mice (Fig. 6a, upper panels). Similarly, alizarin red staining indicated that fewer mineralized nodules were formed in MKO BMSCs (Fig. 6a, lower panels). Consistently, the protein levels of two key osteogenic regulators, RUNX2 and OSTERIX, were decreased in MKO BMSCs during osteogenic differentiation (Fig. 6b). The mRNA levels of the key osteogenic genes (*Runx2*, *Osterix*, *Alpl*, *Ibsp* and *Bglap)* were also decreased in MKO BMSCs after osteogenic induction (Fig. 6c). In contrast, overexpression of CUL4B in BMSCs significantly enhanced ALP activity, mineralized nodule formation and the expression of key osteogenic markers (Fig. 6d–f).

On the other hand, oil red O staining revealed enhanced lipid droplet formation in BMSCs from MKO mice after adipogenic induction (Fig. 6g). PPARγ and C/EBPα levels were elevated significantly in MKO BMSCs during adipogenic differentiation (Fig. 6h). The expression of key adipogenic marker genes, including *Pparg*, *Cebpa*, *Fabp4*, *Adipoq* and *Plin1*, was also upregulated in MKO BMSCs after adipogenic induction (Fig. 6i). In contrast, overexpression of CUL4B in BMSCs inhibited adipogenic differentiation (Fig. 6j–l).

The effect of CUL4B on MSC differentiation was further confirmed in ST2, a bone marrow stromal cell line with the capacity to differentiate into both osteoblasts and adipocytes. Knockdown of *Cul4b* significantly inhibited osteogenic differentiation (Fig. S8a, b) and enhanced adipogenic differentiation (Fig. S8c, d), whereas overexpression of CUL4B in ST2 cells enhanced osteogenic differentiation (Fig. S8e, f) and inhibited adipogenic differentiation (Fig. S8g, h).

Taken together, these results indicate that CUL4B promotes osteogenic differentiation and suppresses adipogenic differentiation of MSCs.

### CUL4B negatively regulates *Klf4* and *Cebpd* expression

**Fig. 7:**
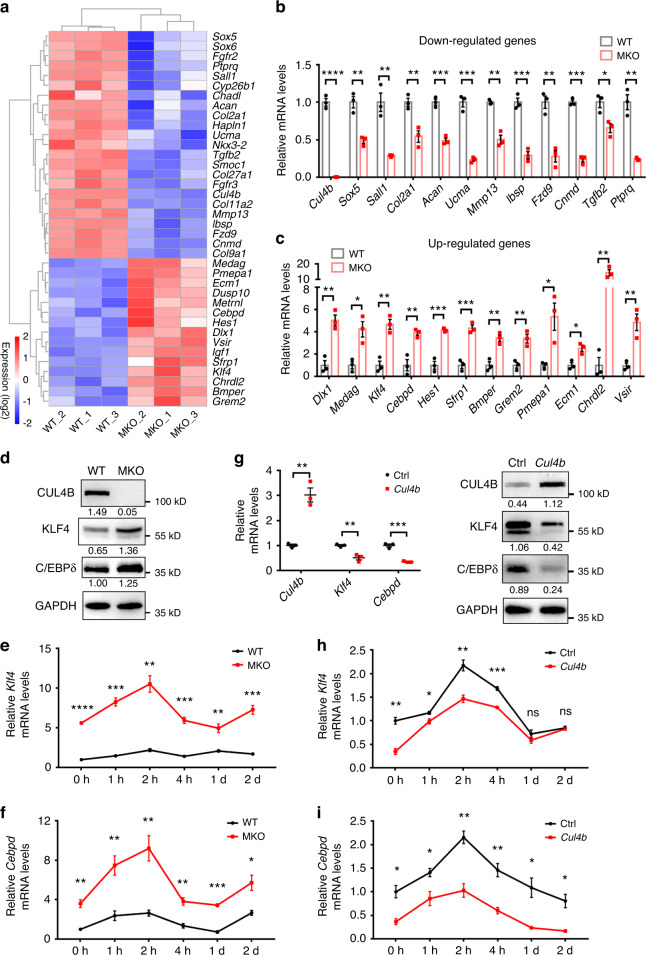
CUL4B negatively regulates *Klf4* and *Cebpd* expression. **a** Heatmap of differentially expressed genes (DEGs) between primary cultured BMSCs from MKO and WT mice. Gene expression values are colored from blue (downregulated) to red (upregulated). **b**, **c** The expression of downregulated (**b**) and upregulated (**c**) DEGs in MKO BMSCs (*n* = 3) was verified by real-time qPCR. **d** The protein levels of KLF4 and C/EBPδ in MKO and WT BMSCs were detected by Western blotting. **e** The mRNA levels of *Klf4* in MKO and WT BMSCs (*n* = 3) during osteogenic differentiation were analyzed by real-time qPCR. **f** The mRNA levels of *Cebpd* in MKO and WT BMSCs (*N* = 3) during adipogenic differentiation were analyzed by real-time qPCR. **g** The mRNA and protein levels of KLF4 and C/EBPδ in CUL4B-overexpressing and control BMSCs (*n* = 3) were analyzed by real-time qPCR and Western blotting. **h** The mRNA levels of *Klf4* in CUL4B-overexpressing and control BMSCs (*n* = 3) during osteogenic differentiation were analyzed by real-time qPCR. **i** The mRNA levels of *Cebpd* in CUL4B-overexpressing and control BMSCs (*n* = 3) during adipogenic differentiation were analyzed by real-time qPCR. Error bars represent standard errors. *P < 0.05; **P < 0.01; ***P < 0.001; ****P < 0.000 1; ns, no significance

To dissect the mechanism underlying CUL4B regulation of fate decisions in MSCs, we performed RNA sequencing (RNA-seq). Pearson correlation coefficients of samples with the same genotypes were greater than 0.92, indicating good correlations between samples (Fig. S9a). A total of 662 differentially expressed genes (DEGs) were identified, of which 457 genes were upregulated and 205 genes were downregulated in MKO BMSCs compared to control BMSCs (Fig. S9b). Cluster analysis of DEGs showed that the expression levels of cartilage and bone matrix components (*Col2a1, Acan*, *Ucma*, *Mmp13, Ibsp*), cartilage and bone development-related genes (*Sox5, Fzd9, Cnmd, Tgfb2, Fgfr2*), stem cell characteristics-related factor (*Sall1*) and fat formation inhibitor (*Ptprq*) were decreased, while the expression levels of osteogenic pathway inhibitors (*Dlx1, Klf4, Hes1, Sfrp1, Bmper, Grem2, Pmepa1, Ecm1*) and adipose differentiation-related factors (*Medag, Klf4, Cebpd*) were increased (Fig. 7a). We validated the expression changes of 23 genes that could be involved in osteogenesis or adipogenesis (Fig. 7b, c). Among these DEGs, we identified *Klf4* and *Cebpd*, which encode two key early-acting osteogenic (KLF4) and adipogenic (C/EBPδ) transcription factors, as our target genes. The expression levels of KLF4 and C/EBPδ were clearly increased in MKO BMSCs (Fig. 7d). Increased *Klf4* mRNA levels were also observed in MKO BMSCs under osteogenic differentiation conditions (Fig. 7e). Similarly, *Cebpd* expression was significantly increased in MKO BMSCs during the course of adipogenic differentiation (Fig. 7f). In contrast, overexpression of CUL4B in BMSCs downregulated the expression of KLF4 and C/EBPδ (Fig. 7g). Additionally, the expression of *Klf4* was decreased in CUL4B-overexpressing BMSCs during osteogenic differentiation (Fig. 7h), and *Cebpd* expression was decreased in CUL4B-overexpressing BMSCs during adipogenic differentiation (Fig. 7i). The above results suggest that CUL4B negatively regulates *Klf4* and *Cebpd* expression.

### CUL4B controls BMSC differentiation by regulating KLF4 and C/EBPδ expression

**Fig. 8:**
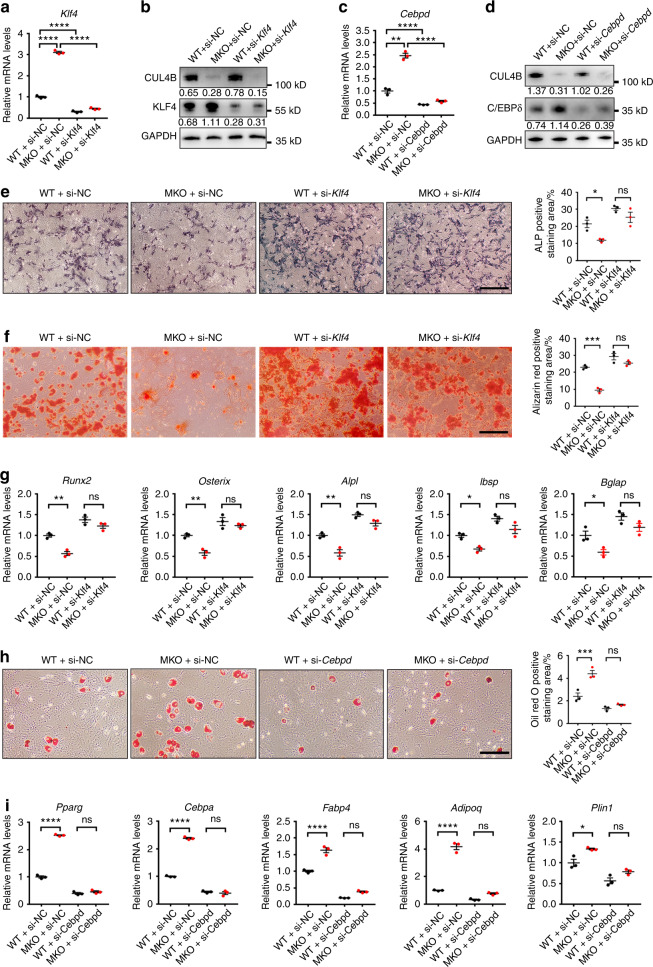
CUL4B controls BMSC differentiation by regulating KLF4 and C/EBPδ expression. **a**, **b** MKO and WT BMSCs (*n* = 3) were transfected with *Klf4* siRNA as well as negative control (NC) siRNA. The mRNA and protein levels of KLF4 were analyzed by qPCR (**a**) and Western blotting (**b**). **c**, **d** MKO and WT BMSCs (*n* = 3) were transfected with *Cebpd* siRNA as well as NC siRNA. The mRNA and protein levels of C/EBPδ were analyzed by qPCR (**c**) and Western blotting (**d**). **e** ALP activity in BMSCs after 7 days of osteogenic induction (*n* = 3). Scale bar, 200 μm. **f** Alizarin red staining showing mineralized nodule formation in BMSCs after 14 days of osteogenic induction (*n* = 3). Scale bar, 200 μm. **g** The expression of key osteogenic marker genes (*Runx2, Osterix, Alpl, Ibsp* and *Bglap*) in BMSCs after osteogenic induction was detected by qPCR (*n* = 3). **h** Oil red O staining showing lipid accumulation in BMSCs after 10 days of adipogenic induction (*n* = 3). Scale bar, 100 μm. **i** The expression of key adipogenic marker genes (*Pparg, Cebpa, Fabp4, Adipoq* and *Plin1*) in BMSCs after adipogenic induction was detected by qPCR (*n* = 3). Error bars represent standard errors. *P < 0.05; **P < 0.01; ***P < 0.001; ****P < 0.000 1; ns, no significance

To test whether upregulated KLF4 and C/EBPδ expression by CUL4B depletion is responsible for altered MSC differentiation, we used siRNA to interfere with *Klf4* or *Cebpd* expression in control and MKO BMSCs, respectively. The knockdown of KLF4 or C/EBPδ by siRNA was confirmed by qPCR and Western blotting (Fig. 8a–d). The decreased ALP activity and mineralized nodule formation in MKO BMSCs after osteogenic induction were fully rescued by interfering with *Klf4* expression (Fig. 8e, f). Consistently, the decreased expression of *Runx2*, *Osterix*, *Alpl*, *Ibsp* and *Bglap* in MKO BMSCs after osteogenic induction was restored by knocking down *Klf4* (Fig. 8g). These results suggest that upregulated KLF4 expression in MKO BMSCs is responsible for the decreased osteogenic differentiation induced by CUL4B depletion. Similarly, the increased lipid accumulation in MKO BMSCs after adipogenic induction was fully rescued by interfering with *Cebpd* expression (Fig. 8h). The increased expression of key adipogenic marker genes (*Pparg*, *Cebpa*, *Fabp4*, *Adipoq* and *Plin1*) in MKO BMSCs after adipogenic induction was attenuated by knockdown of *Cebpd* (Fig. 8i), suggesting that loss of CUL4B increases adipogenic differentiation of BMSCs through upregulation of *Cebpd* expression. The above results indicate that elevated expression of *Klf4* and *Cebpd* contributes to enhanced adipogenic and decreased osteogenic differentiation in CUL4B-deficient BMSCs.

### CUL4B epigenetically represses *Klf4* and *Cebpd* transcription

**Fig. 9:**
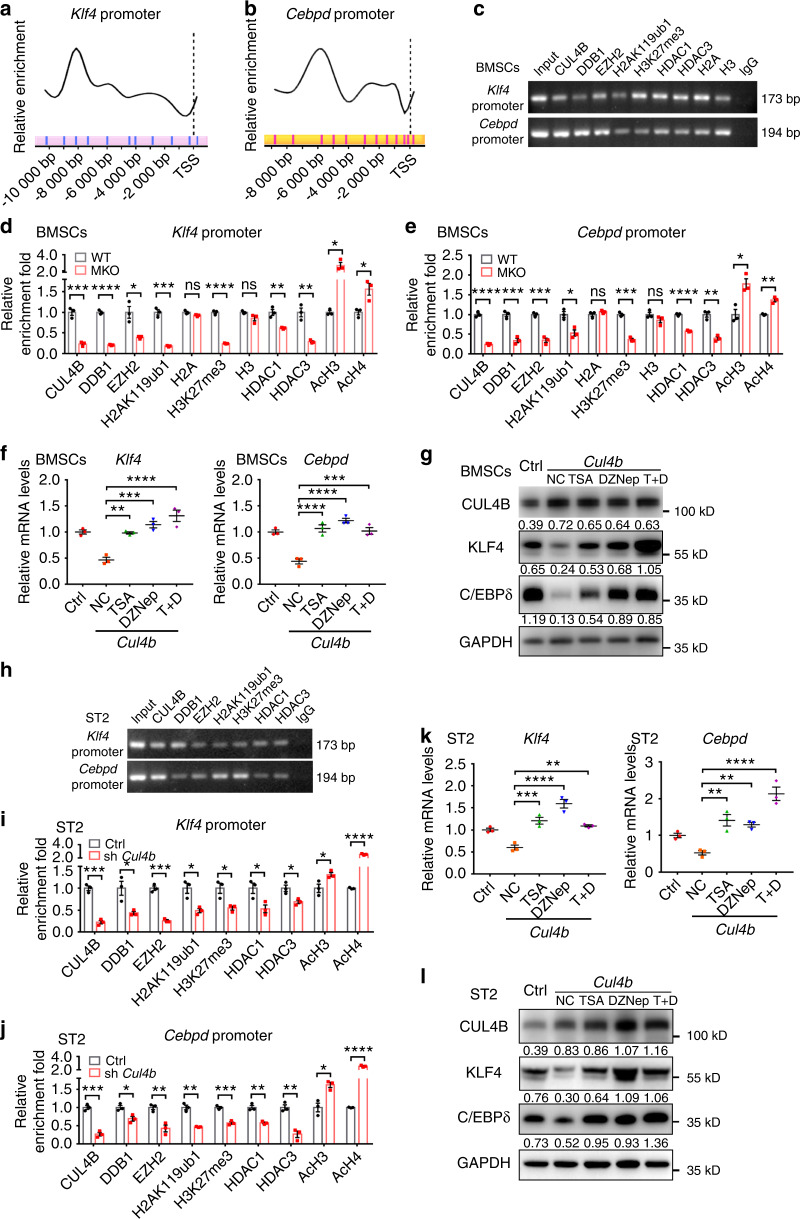
CUL4B epigenetically represses *Klf4* and *Cebpd* transcription. **a**, **b** qChIP assays were conducted to determine the binding regions for CUL4B at the promoters of *Klf4* (**a**) and *Cebpd* (**b**) in BMSCs. The results are represented as the fold change over the control (IgG). **c** ChIP assays using BMSCs showed that CUL4B, DDB1, EZH2, H2AK119ub1, H3K27me3, HDAC1 and HDAC3 were enriched at the promoters of *Klf4* and *Cebpd*. **d**, **e** qChIP assays of the recruitment of the indicated proteins at the *Klf4* (**d**) and *Cebpd* (**e**) promoters in MKO and WT BMSCs (*n* = 3). H2A and H3 were used as controls. **f**, **g** Expression of *Klf4* and *Cebpd* in CUL4B-overexpressing BMSCs (*n* = 3) treated with TSA and/or DZNep was detected by real-time qPCR (**f**) and Western blotting (**g**). **h** ChIP assays using ST2 cells showed that CUL4B, DDB1, EZH2, H2AK119ub1, H3K27me3, HDAC1 and HDAC3 were enriched at the promoters of *Klf4* and *Cebpd*. **i**, **j** qChIP assays of the recruitment of the indicated proteins at the *Klf4* (**i**) and *Cebpd* (**j**) promoters in control and *Cul4b* knockdown ST2 cells (*n* = 3). **k**, **l** Expression of *Klf4* and *Cebpd* in CUL4B-overexpressing ST2 cells (*n* = 3) treated with TSA and/or DZNep was detected by real-time qPCR (**k**) and Western blotting (**l**). Error bars represent standard errors. *P < 0.05; **P < 0.01; ***P < 0.001; ****P < 0.000 1; ns, no significance

The CRL4B complex can catalyze H2AK119 monoubiquitination and coordinate with the PRC2 complex or SIN3A/HDAC complex to epigenetically repress target genes.^22,23^ To determine whether the CRL4B complex can directly repress *Klf4* and *Cebpd* expression, we performed quantitative chromatin immunoprecipitation (qChIP) assays using primers covering the ~10 kb region upstream of the transcription start site (TSS) of *Klf4* and *Cebpd*. CUL4B directly bound to the region between −8 255 bp and −8 083 bp upstream of the *Klf4* TSS and the region between −5 838 bp and −5 645 bp upstream of the *Cebpd* TSS (Fig. 9a, b). Notably, DDB1 (another important component of the CRL4B complex), EZH2 (core component of the PRC2 complex), H2AK119ub1, H3K27me3, HDAC1 and HDAC3 were also enriched in the same region (Fig. 9c). We next evaluated the effect of CUL4B depletion on the recruitment of DDB1, EZH2, HDAC1 and HDAC3 to the promoters of *Klf4* and *Cebpd*. The results showed that deletion of *Cul4b* significantly reduced the enrichment of DDB1, EZH2, HDAC1 and HDAC3 in the promoters of *Klf4* and *Cebpd*. Consistently, H2AK119ub1 and H3K27me3 levels in the promoters of *Klf4* and *Cebpd* were significantly decreased by loss of CUL4B, while acetylated histones H3 and H4 levels were significantly increased (Fig. 9d, e). Furthermore, treatment with DZNep (EZH2 inhibitor) and/or TSA (HDAC inhibitor) efficiently blocked the reduction in *Klf4* and *Cebpd* expression caused by CUL4B overexpression (Fig. 9f, g), suggesting that CUL4B regulation of *Klf4* and *Cebpd* expression relies on the activity of PRC2 and HDACs. Similar results were found in ST2 cells (Fig. 9h–l). Taken together, these results reveal that the CRL4B complex cooperates with the PRC2 complex and HDACs to repress the transcription of *Klf4* and *Cebpd* by promoting H2AK119 monoubiquitination, H3K27 trimethylation, and H3 and H4 deacetylation in the promoter regions.

## Discussion

**Fig. 10:**
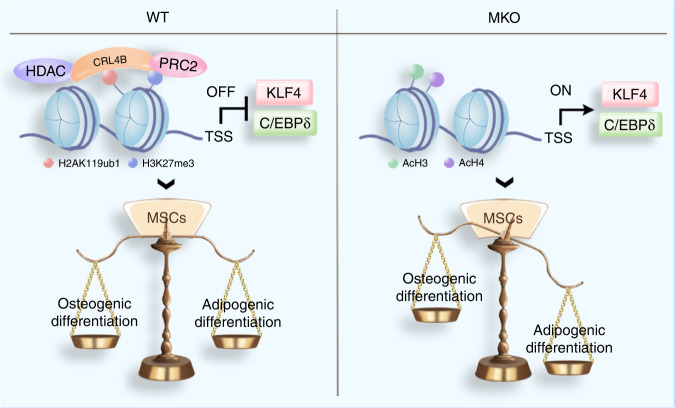
A model by which CUL4B regulates MSC fate commitment. Left, high levels of CUL4B in BMSCs of young mice could repress the transcription of *Klf4* and *cebpd* and sustain osteogenic rather than adipogenic differentiation. Right, loss of CUL4B in BMSCs of MKO mice or decreased expression of CUL4B in BMSCs of old mice leads to increased expression of *Klf4* and *cebpd* and promotes adipogenic differentiation at the expense of osteogenic differentiation

Our results revealed that CUL4B orchestrates MSC commitment during bone development and skeletal aging. Under aging or pathological stimuli, BMSCs favor adipogenic differentiation to osteogenic differentiation, resulting in reduced bone formation and increased MAT accumulation, causing osteoporosis.^24,25^ We found that the expression of CUL4B in BMSCs decreased with aging in both humans and mice. Using a conditional knockout mouse model, we showed that depletion of CUL4B in MSCs exacerbated age-related and estrogen deficiency-induced bone loss and MAT accumulation. Depletion of CUL4B in MSCs also reduced bone strength. Our in vitro gain- and loss-of-function studies further confirmed that CUL4B intrinsically promotes osteogenic differentiation and restrains adipogenic differentiation. Our data suggest a model in which high levels of CUL4B in young MSCs make them favor bone formation over adipogenesis, which promotes the physiological balance (Fig. 10, left). In aged MSCs (or in MKO mice), a decline in CUL4B levels alleviated the repression of *Klf4* and *Cebpd* transcription to promote the adipogenic differentiation of MSCs at the expense of osteogenic differentiation (Fig. 10, right).

Depletion of CUL4B in MSCs also causes postnatal skeletal development defects, in addition to exacerbated age-related bone loss. The impaired development of the growth plates leads to long bone shortening in MKO mice, which recapitulates the short stature phenotype in human patients with *CUL4B* mutation. These results suggest that CUL4B plays important roles in both skeletal development and age-related bone loss. Further studies using tamoxifen-inducible *Cre* to restrain Cul4b depletion to specific ages will help clarify how much the developmental defects contribute to the phenotypes in aged animals.

Our work also shows that deletion of CUL4B in MSCs leads to increased osteoclasts. As increased bone resorption is critical for bone loss, attention should be given to the effect of CUL4B depletion in MSCs on osteoclasts. Since *Cul4b* was not deleted in monocytes and/or macrophages (Fig. 2b), the precursors of osteoclasts, we hypothesized that depletion of CUL4B in MSCs and their progeny may indirectly affect osteoclast differentiation. Osteoblasts regulate osteoclast function through direct cell-to-cell contact or through secretory proteins^26^; thus, this intriguing phenomenon warrants further investigation by selective deletion of CUL4B in the osteoblast lineage.

KLF4 is a key MSC fate determinant factor. KLF4 attenuates osteoblast formation by interacting directly with RUNX2 and repressing the expression of its target genes.^11^ KLF4 is highly expressed in immature osteoblasts but diminishes in mature osteoblasts.^27,28^ However, the upstream regulation of KLF4 expression remains unclear. Our studies identified CUL4B as an epigenetic repressor of KLF4. The skeletal phenotypes of *Prx1-Cre;Cul4b*^*flox/Y*^ mice were similar to those of *Klf4* transgenic mice, which exhibited disturbed membranous and endochondral ossifications.^29^ Importantly, knockdown of *Klf4* expression restored the osteogenic differentiation of *Cul4b*-deficient BMSCs. Thus, we conclude that augmentation of KLF4 induced by CUL4B depletion contributes to decreased osteogenesis.

C/EBPs are key regulators of adipocyte differentiation. Both C/EBPβ and C/EBPδ are induced during early adipogenic differentiation but decrease at late stages and are replaced by PPARγ and C/EBPα.^30,31^ The C/EBPβ and C/EBPδ-null mice showed severely impaired adipogenesis.^32^ Our study showed that CUL4B repressed C/EBPδ expression and that knockdown of C/EBPδ expression suppressed the elevated adipogenic differentiation caused by loss of CUL4B. Of note, KLF4 is also an essential early regulator of adipogenesis by transcriptional activation of C/EBPβ.^12^ Thus, increased KLF4 may also facilitate the enhanced adipogenesis induced by CUL4B deficiency.

In addition to *Klf4* and *Cebpd*, other target genes might be involved in the regulation of MSC differentiation by CUL4B. For example, *Chrdl2*, an antagonist of bone morphogenetic protein (BMP), prevents BMPs from interacting with their cell surface receptors.^33^ *Fzd9* knockout mice exhibited low bone mass and decreased bone formation.^34^ *Medag* is an adipogenic gene, and *Medag* overexpression promotes preadipocyte differentiation and lipid accumulation as well as increases glucose uptake in adipocytes.^35^ Overexpression of *Ptprq* led to reduced differentiation of MSCs into adipocytes.^36^ The regulatory network is complex and needs to be further characterized.

Epigenetic factors play critical roles in MSC commitment.^37,38^ MSC differentiation is extensively coordinated by a network of transcription factors and epigenetic factors.^39,40^ Studies from our group and others have established CUL4B as an important epigenetic regulator. The CRL4B complex catalyzes H2AK119 monoubiquitination. It can coordinate with the PRC2 complex to promote H3K27 trimethylation,^22^ with SUV39H1/HP1/DNMT3A to facilitate H3K9 trimethylation and DNA methylation,^41^ or with the DIN3A-HDAC complex to deacetylate H3 and H4.^23^ KLF4 expression in cancer and stem cells has been shown to be modulated by epigenetic control, such as DNA methylation and histone methylation.^42,43^ Here, we demonstrate that CRL4B binds to the promoters of *Klf4* and *Cebpd* and epigenetically represses *Klf4* and *Cebpd* transcription through coordination with EZH2 and HDAC1/3.

In summary, our studies establish a critical role of CUL4B in orchestrating MSC commitment. Lack of CUL4B in MSCs impairs postnatal skeletal development and aggravates bone loss and MAT accumulation during aging or after ovariectomy, suggesting that CUL4B functions as an osteoprotective factor to promote osteogenesis and inhibit adipogenesis. Our findings have important clinical significance in clarifying the pathological mechanism and developing new therapies for bone-related diseases.

## Materials and Methods

### Mice

Female *Cul4b*^*flox/flox*^ mice, which were generated as previously reported,^44^ were crossed with male *Prx1*-*Cre* mice to generate conditional knockout mice. As the *Cul4b* gene is located on the X chromosome, we obtained male *Cul4b* conditional knockout mice (*Prx1*-*Cre*^*+/−*^; *Cul4b*^*flox/Y*^), male wild-type mice (*Prx1*-*Cre*^*−/−*^;*Cul4b*^*flox/Y*^), female heterozygous mice (*Prx1*-*Cre*^*+/−*^; *Cul4b*^*flox/+*^), and female wild-type mice (*Prx1*-*Cre*^*−/−*^;*Cul4b*^*flox/+*^) among the offspring. Littermate *Prx1*-*Cre*^*−/−*^;*Cul4b*^*flox/Y*^ mice were used as controls (WT). All analyses were performed on male mice except for OVX mice. *Prx1*-*Cre*^*+/−*^; *Cul4b*^*flox/Y*^ mice were further bred to *Cul4b*^*flox/flox*^ mice to produce male *Cul4b* conditional knockout mice (*Prx1*-*Cre*^*+/−*^; *Cul4b*^*flox/Y*^), male wild-type mice (*Prx1*-*Cre*^*−/−*^;*Cul4b*^*flox/Y*^), female *Cul4b* conditional knockout mice (*Prx1*-*Cre*^*+/−*^; *Cul4b*^*flox/flox*^) and female wild-type mice (*Prx1*-*Cre*^*−/−*^;*Cul4b*^*flox/flox*^). Mice underwent sham or OVX operation at 3 months and were euthanized after 1 month. The mice were on the C57BL/6 J background. All animal care and experiments were approved by the Animal Care and Use Committee of the School of Basic Medical Sciences of Shandong University (No. ECSBMSSDU2018-2-004).

### Micro-CT and radiography

Mouse femurs were scanned using a micro-CT (SkyScan1176 in-vivo Micro-CT, BRUKER, Kontich, Belgium), and CT Analyzer Skyscan software was used to analyze the data and produce three-dimensional reconstruction images. Radiographic images were obtained using micro-CT’s built-in X-ray radiography system, and shooting parameters were as follows: 9 μm voxel size, 50 kV, 500 μA, 1 000 ms exposure time, Al 0.5 mm filter, 0.7° rotation step.

### Histological Analysis

The femur and tibia were dissected, fixed in 4% formaldehyde at 4 °C for 24 h and decalcified in 15% EDTA (pH 7.4) at 4 °C for 2 weeks. Then, the tissues were dehydrated, embedded in paraffin and sectioned at a thickness of 4 μm for H&E staining, safranin O staining, TRAP staining and immunostaining.

### Immunostaining and bone histomorphometry

Immunofluorescence (IF) and immunohistochemistry (IHC) were performed as previously described in ref. ^45^. Primary antibodies included anti-Nestin (Proteintech, 19483-1-AP, 1:200), anti-CUL4B (Sigma, C9995, 1:200), anti-OCN (Abcam, ab93876, 1:200) and anti-FABP4 (Abcam, ab92501, 1:200). Secondary antibodies included goat anti-rabbit or mouse horseradish peroxidase (HRP) and goat anti-rabbit or mouse Cy2 or Cy3 (Jackson ImmunoResearch, 1:200). Integral optical density (IOD) in IHC was quantified by ImageJ.

### Double fluorochrome labeling

Double fluorescence labeling was performed as previously described in ref. ^45^. Briefly, alizarin red (20 mg·kg^−1^ i.p.; Sigma‒Aldrich) and calcein (5 mg·kg^−1^ i.p.; Sigma‒Aldrich) were administered to mice on Day 1 and Day 8, respectively. Mice were euthanized on Day 10. The nondecalcified femur tissues were dehydrated, embedded in methyl methacrylate, and sectioned. The unstained sections were photographed using a fluorescence microscope (Olympus).

### Serology

For analysis of serum OCN and Trap5b, blood was collected by heart puncture and centrifuged at 6 500 r·min^−1^ for 5 min. The serum OCN levels were measured using a Mouse OC/BGP (Osteocalcin) ELISA Kit (Elabscience). The serum Trap5b levels were measured using a Trap5b kit (Njjcbio). All serum samples were assayed in duplicate.

### Three-point bending test

The femurs were subjected to the three-point bending test using an HY-1080 Computer Controlled Electronic Universal Material Testing Machine at room temperature of 23 °C. The femurs were oriented such that the anterior-posterior axis was in the loading direction, while the medial-lateral axis was in the bending direction. The femur loaded is shown in Fig. 3a. For the femurs, a span length of 6 mm was utilized. The bones were positioned at the center of the supports and subjected to a vertical force at a constant speed of 3 mm·min^−1^ directed toward the midshaft until they fractured. Load and displacement were recorded by M213 Pro Mechanical Testing Machine Test Software. Then, the load‒displacement curve was plotted by Origin 2021b Software. The biomechanical properties were assessed through the three-point bending test (detailed in Supplemental Material).

### Primary BMSC culture

BMSCs were isolated from mouse compact bone and cultured.^46^ Briefly, mice were euthanized, and long bones were dissected. The epiphyses were cut off, and the bone cavities were washed. The compact bones were excised into chips of 1–2 mm^3^, cultured in a 6 cm^2^ cell culture dish and digested by collagenase II (Worthington). Finally, the bone chips were cultured at 37 °C for 3 days and digested by trypsin (Gibco) for passage. Passage 3–5 cells were used for experiments unless otherwise described. For the generation of BMSCs that overexpress CUL4B, the pcDNA3-CUL4B vector or empty pcDNA3 vector was transfected into BMSCs using Lipo3000 reagent (Thermo).

### Osteogenic and adipogenic differentiation

Passage 3–5 BMSCs or ST2 cells (BeNa Culture Collection) were induced with osteogenic media (OriCell) containing α-MEM, 10% FBS, L-glutamine, β-glycerophosphate, ascorbic acid, dexamethasone and penicillin/streptomycin. ALP activity and alizarin red staining were performed after 7 or 14 days of osteogenic induction, respectively.

Passage 3–5 BMSCs or ST2 cells were induced with adipogenic media containing α-MEM, 10% FBS, isobutylmethylxanthine, dexamethasone and insulin. Oil red O staining was performed after 10 days of adipogenic induction using a commercial staining solution (Sangon).

### Western blotting

Western blotting was performed as previously described in ref. ^45^. The primary antibodies included CUL4B (Sigma, C9995), RUNX2 (CST, 12556 S), OSTERIX (Abcam, ab209484), PPARγ (CST, 2435 S), C/EBPα (CST, 8178 T), KLF4 (CST, 4038 T), C/EBPδ (CST, 2318 T) and GAPDH (Abmart, M20006S). These antibodies were used at a 1:1 000 dilution. Secondary antibodies included anti-rabbit or anti-mouse horseradish peroxidase (HRP) (Abmart). The membranes were subjected to chemiluminescence detection (Thermo) and then exposed by an Amersham Imager 600 (GE) to visualize the bands. Estimates of protein amounts were obtained by measuring the area of protein bands using ImageJ and were normalized to GAPDH protein amount in the respective samples.

### Quantitative real-time RT‒PCR (qPCR)

Total RNA from MKO and control BMSCs was extracted using TRIzol reagent (Invitrogen). Freshly isolated RNA was reverse transcribed to generate cDNA. The resulting cDNA was then subjected to quantitative real-time RT‒PCR using 2×ChamQ SYBR Color qPCR Master Mix (Vazyme). The quantified individual RNA expression levels were normalized to *Gapdh*. The sequences of the primers are listed in Table S1.

### RNA-seq

A total amount of 1 µg RNA per sample was used for sample preparations. A NEBNext® Ultra^TM^ RNA Library Prep Kit (Illumina) was used to generate sequencing libraries. After purification, all libraries were sequenced on Illumina HiSeq X Ten (Illumina, San Diego, CA, USA) to acquire 150 bp paired-end sequence reads at Novogene Corporation. Three replicates of BMSCs for each group were sequenced. Differential expression analysis was performed utilizing the DESeq2 R package (1.16.1). Genes with an adjusted *P*-value less than 0.05 were considered differentially expressed.

### siRNA interference

Primary BMSCs seeded on 6-well plates were transfected with 15 nmol·L^−1^ siRNA using Lipofectamine RNAiMAX reagent (Thermo). si-NC, si-*Klf4* and si-*Cebpd* were customized at RiboBio Company. The siRNA oligonucleotide sequences were 5’-GCAGCTTGCAGCAGTAACA-3’ *for Klf4 and* 5’-ACTTCAGCGCCTACATTGA-3’ for *Cebpd*.

### Chromatin immunoprecipitation (ChIP) assay

ChIP assays were performed following the manufacturer’s recommendations (CST). Briefly, cells were crosslinked, sonicated, precleared, and incubated with antibody. Then, DNA was purified and analyzed by conventional PCR or quantitative real-time PCR. The antibodies were anti-CUL4B (Sigma, C9995), anti-DDB1 (Proteintech, 11380-1-AP), anti-EZH2 (CST, 5246 S), anti-HDAC1 (Abcam, ab280198), anti-HDAC3 (CST, 85057 S), anti-H2AK119ub1 (CST, 8240 S), anti-H3K27me3 (Abcam, ab6002), anti-AcH3 (CST, 8173 S), anti-AcH4 (Abcam, ab45166), anti-H2A (Proteintech, 10856-1-AP), anti-H3 (CST, 5327 T) and normal rabbit IgG (CST, 2729 P). The sequences of the primers are listed in Table S2.

### Statistical Analysis

Data are expressed as the mean ± SEM. Unpaired Student’s t-test was used to analyze data from two groups for any significant differences, and one-way ANOVA with Bonferroni’s post hoc analysis was used for multiple comparisons when the data involved three or more groups. A *P*-value of less than 0.05 was considered statistically significant. (*P < 0.05, **P < 0.01, ***P < 0.001, ****P < 0.000 1).

## Supplementary information

Supplementary Materials

## Data Availability

Raw and processed data for RNA-seq can be obtained from the BioProject database under accession number PRJNA846938.
