# Supplementary material for: CUL4B orchestrates mesenchymal stem cell commitment by epigenetically repressing KLF4 and C/EBPδ
Source: Bone Res. 2023 Jun 2;11:29. doi: 10.1038/s41413-023-00263-y (PMC10238510; doi:10.1038/s41413-023-00263-y)
Supplement: Supplementary file 1 — Supplementary Materials [file 41413_2023_263_MOESM1_ESM.docx]

Supplementary Materials

**Materials and Methods**

**PCR genotyping**

Genomic DNA was extracted from a tail biopsy and used for genotyping by PCR analysis. The sequences of the primers were for Cre: 5'-CCCGCAGAACCTGAAGATG-3' and 5'-GACCCGGCAAAACAGGTAG-3', and for loxp: 5'-ACAGGTATTTGCCAGTGCTGTC-3' and 5'-TTCTGTTACCTTCCTACCGAGAG-3'.

**Flow cytometric analysis**

The third generation of primary BMSCs were stained with anti-CD29 (BioLegend: 102205), anti-CD44 (eBioscience: 120441), anti-CD105 (eBioscience: 17-1051-80), anti-Sca-1 (BioLegend: 122525), anti-CD45 (eBioscience: 250451), anti-CD11b (eBioscience: 110112), anti-CD14 (BioLegend: 123317) and anti-CD34 (eBioscience: 11-0341-82) antibodies. Data were analyzed with the FlowJo 7.6.5 software.

**Whole mount skeletal staining**

Newborn pups were euthanized. Skeletons were dissected and fixed overnight in 95% ethanol. Then the skeletons were transferred to acetone for defatting for one day. Cartilage elements were stained with a 30% alcian blue dye (dissolved in 80 ml 95% ethanol and 20 ml glacial acetic acid). Skeletons were washed twice with 95% ethanol and then placed in 1% KOH until the remaining soft tissues were dissolved. Bones were stained with 75 mg/ml alizarin red S (Sigma) in 1% KOH overnight and then were placed in a gradient of glycerol containing different concentrations of 1% KOH for two weeks, with daily solution changes. Skeletons were stored in 100% glycerol solution indefinitely.

**Von Kossa staining**

The nondecalcified femur tissues were dehydrated, embedded in methyl methacrylate, and sectioned. The slides were performed with Von Kossa staining following the manufacturer's protocol (Servicebio).

**Biomechanical parameters**

Yield load (YL) refers to the max force that a bone can withstand while remaining elastic, which is obtained from the end of the elastic phase. Maximum load (ML) is obtained from the max value in the load-displacement curve. The stiffness (k) is obtained by calculating the slope of the elastic or quasi-elastic section of the curve, shown in Fig. 5B.

Elasticity modulus of the bone was calculated by the bone dimensions and three-point bending data. Elasticity modulus (1) is defined as the bone’s ability to maintain elastic deformation to an applied force where k is the stiffness, l is the span length, and I is the moment of inertia (2). The femurs were treated as elliptical tubes when calculating moment of inertia, which was calculated by ImageJ using the actual loading orientation, shown in Fig. 5C.

$E=\frac{kl^{3}}{48I}$(1)

$I=\frac{\pi}{4}({a^{'}b}^{'3}-\mathrm{ab}^{3})$(2)

The strength essentially reflects the stress on the bone. Yield Strength (3) represents the stress that a bone can withstand slight plastic deformation.

$YS=\frac{\mathrm{YL}}{\pi(a'b'-ab)}$(3)

Bending strength (4) refers to the maximum stress that a bone can withstand before fracture.

$BS=\frac{\mathrm{ML}}{\pi(a'b'-ab)}$(4)


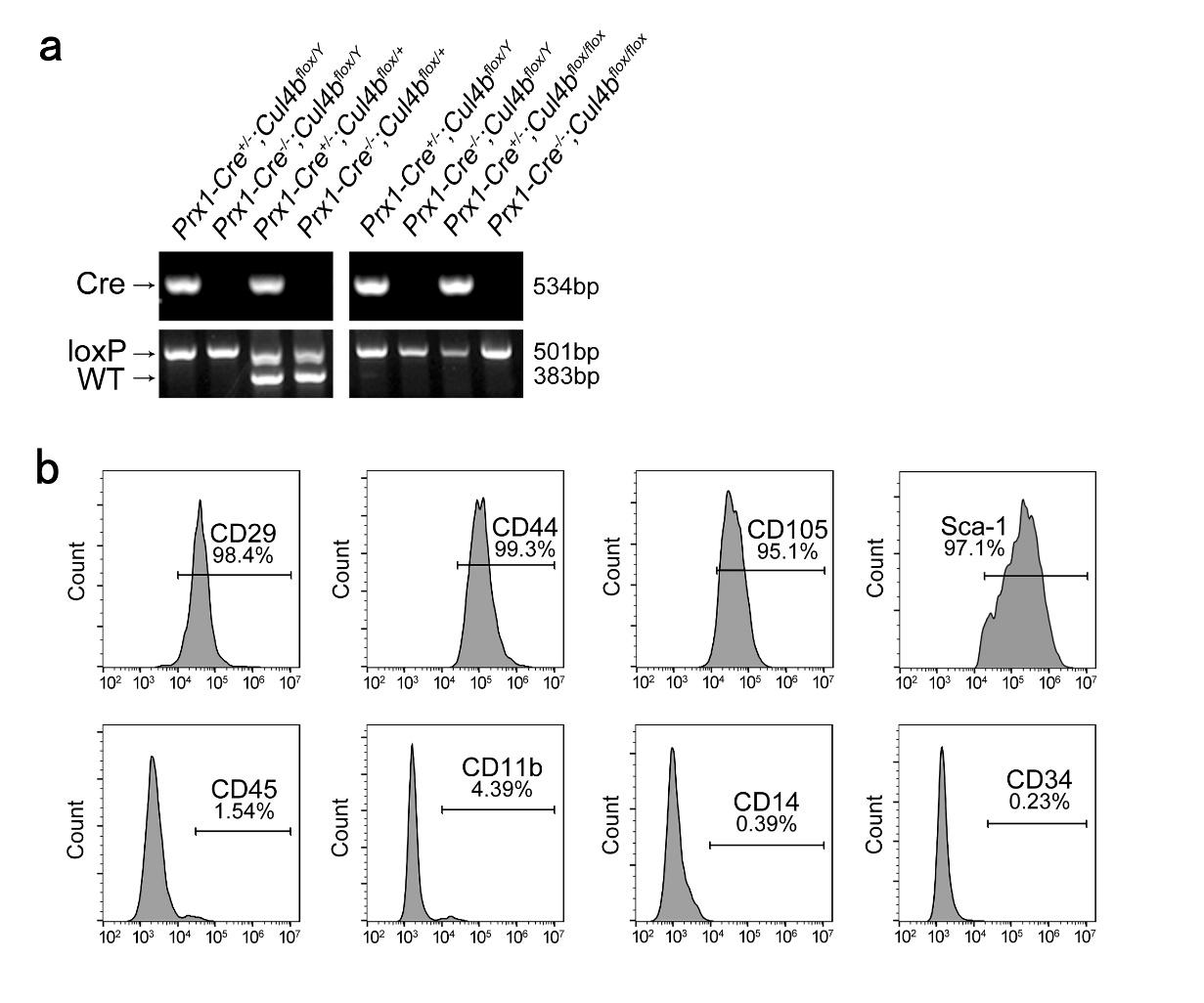


**Fig. S1. Generation of *Cul4b* conditional knockout mice and identification of BMSCs.** (a) The genotypes of mice were identified by PCR. (**b**) Cell surface receptors CD29, CD44, CD105, Sca-1, CD45, CD11b, CD14 and CD34 were detected by flow cytometry to identify BMSCs.


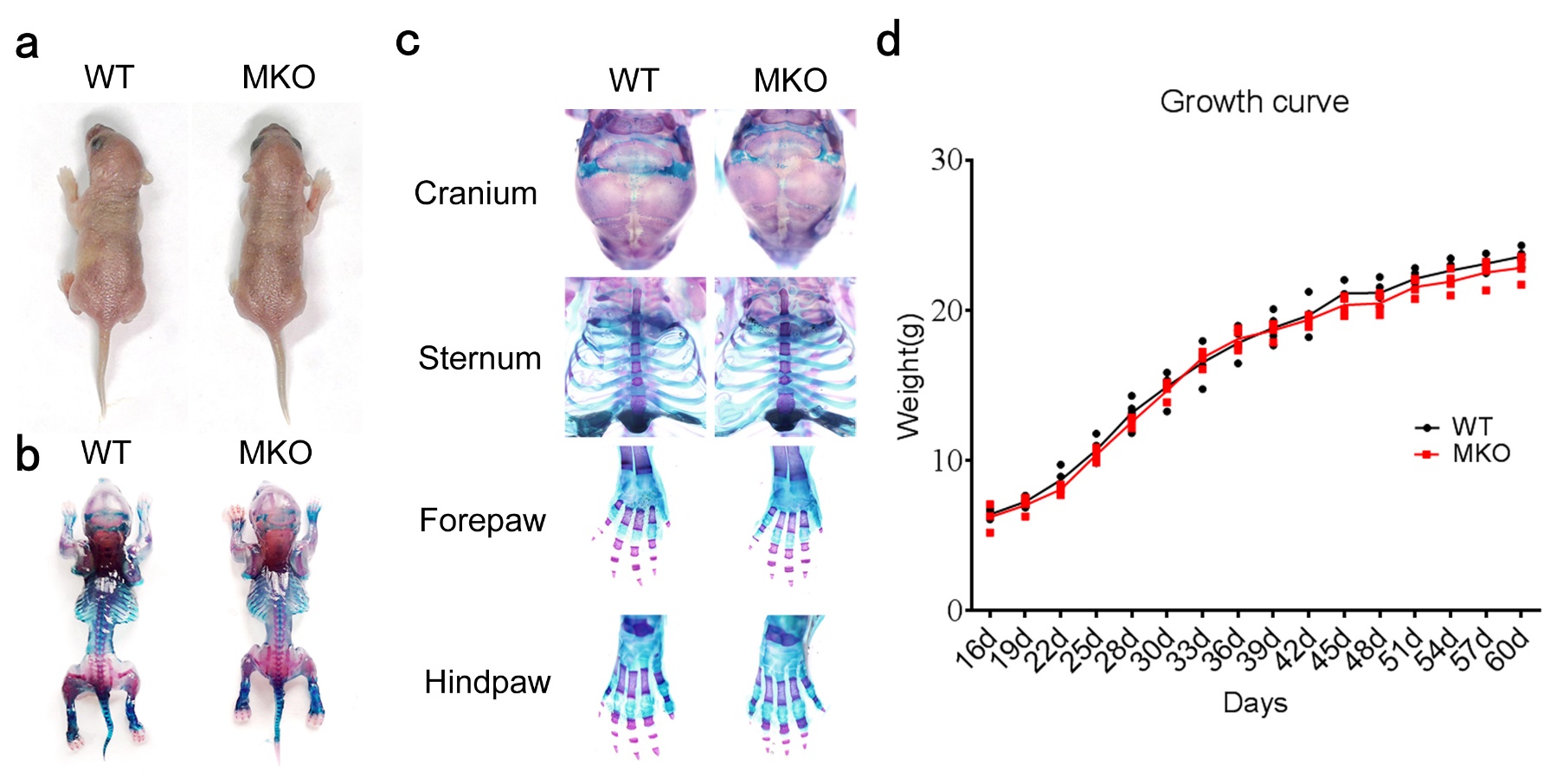


**Fig. S2. Whole mount skeletal staining of newborn mice.** (**a**) Gross appearance of newborn MKO and littermate control mice. (**b**) Whole mount skeletal staining of MKO newborns and littermate controls by alician blue and alizarin red. (**c**) High magnification images of cranium, sternum, forepaw and hindpaw.


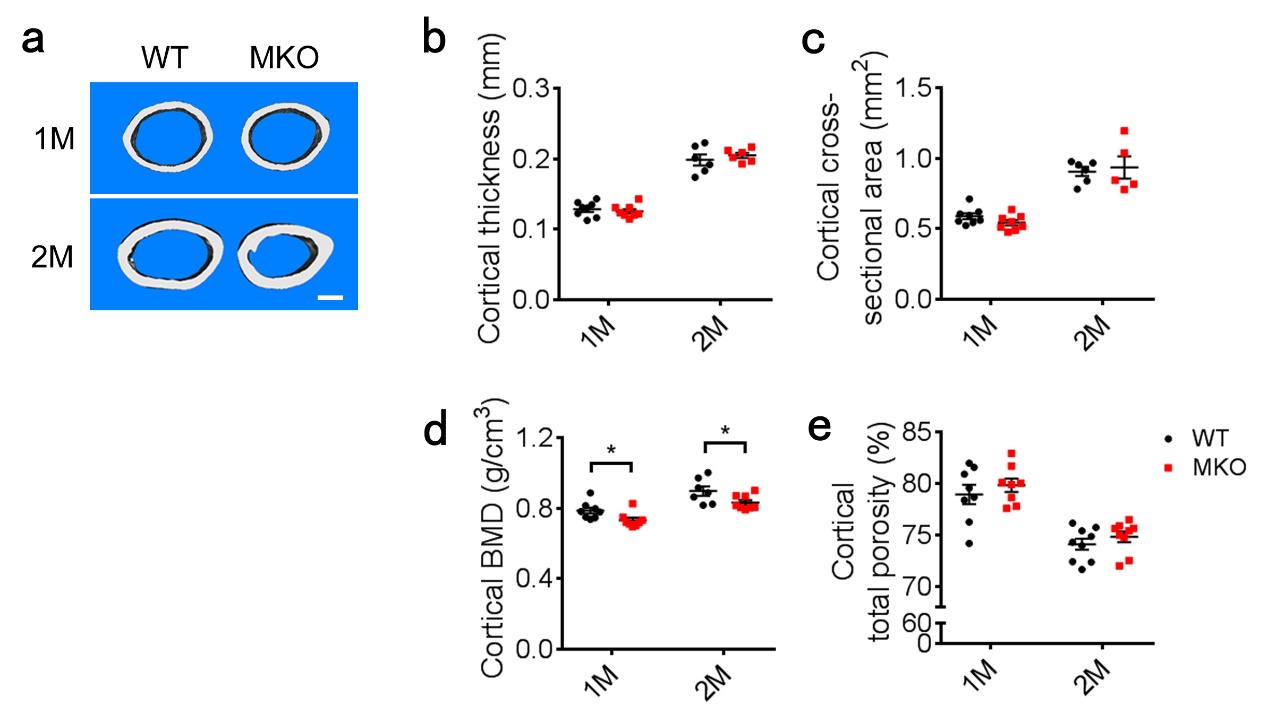


**Fig. S3. Micro-CT analysis of cortical bone in 1 and 2 months.** (**a**) Representative micro-CT three-dimensional reconstruction images of cortical bone of femurs from 1- and 2-month-old WT and MKO mouse. Scale bar, 0.5 mm. (**b**) Quantification of cortical thickness of femurs from 1- (n=8;8) and 2-month-old (*n*=6;6) WT and MKO mouse. (**c**) Quantification of cortical cross-sectional area of femurs from 1- (n=8;8) and 2-month-old (n=6;5) WT and MKO mouse. (**d**) Quantitative measurements of the cortical bone mineral density (BMD) of femurs from 1- (n=8;8) and 2-month-old (*n*=7;8) WT and MKO mice by micro-CT. (**e**) Quantitative measurements of the cortical total porosity of femurs from 1- (n=8;8) and 2-month-old (n=9;9) WT and MKO mouse by micro-CT. Error bars represent standard errors. **p*<0.05.


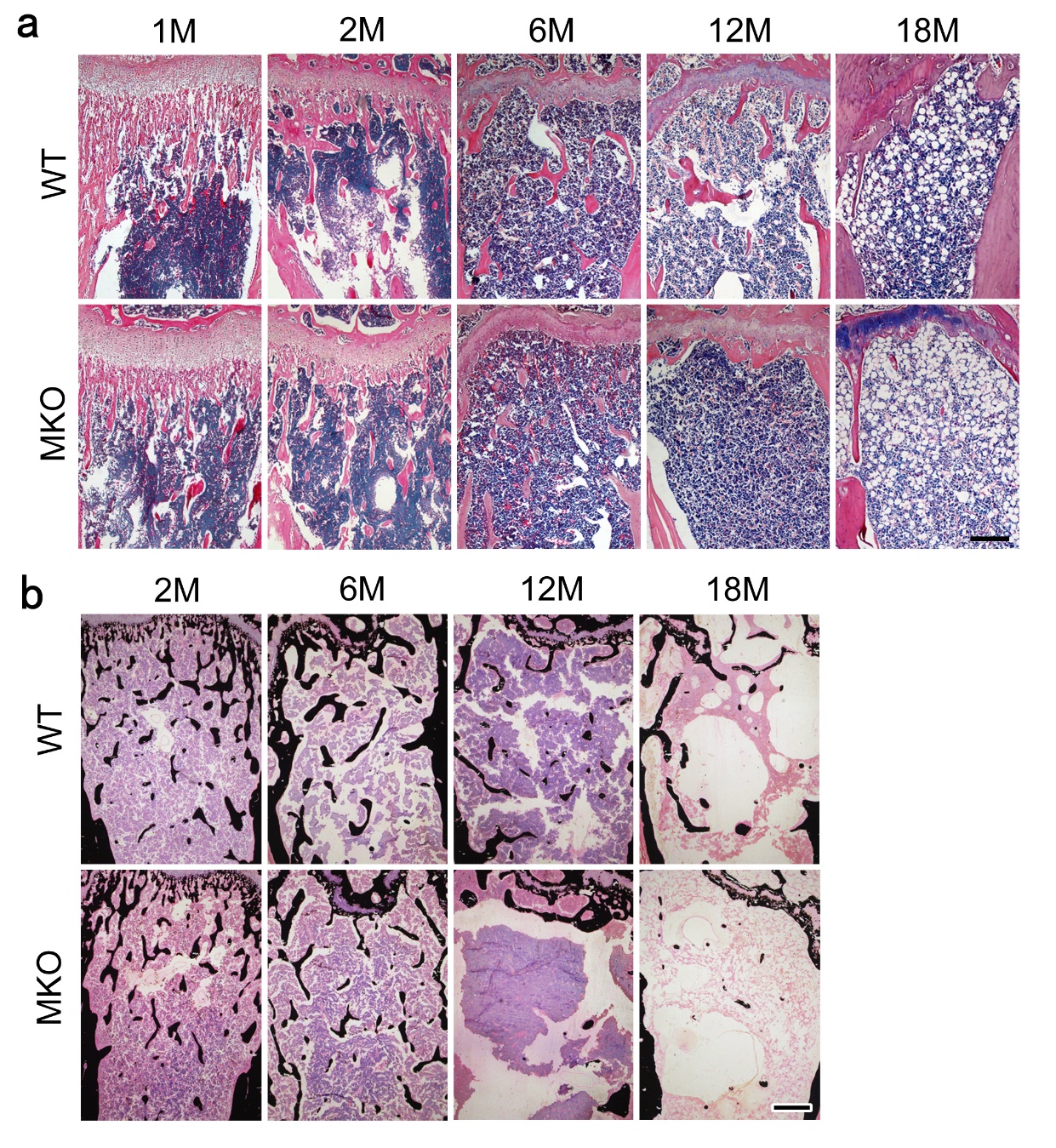


**Fig. S4. Histological analysis of WT and MKO mice.** (**a**) Representative H&E staining of 1-, 2-, 6-, 12- and 18-month-old mouse tibias. Scale bar, 150 μm. (**b**) Representative von Kossa staining of 2-, 6-, 12- and 18-month-old mouse femurs. Scale bar, 200 μm.


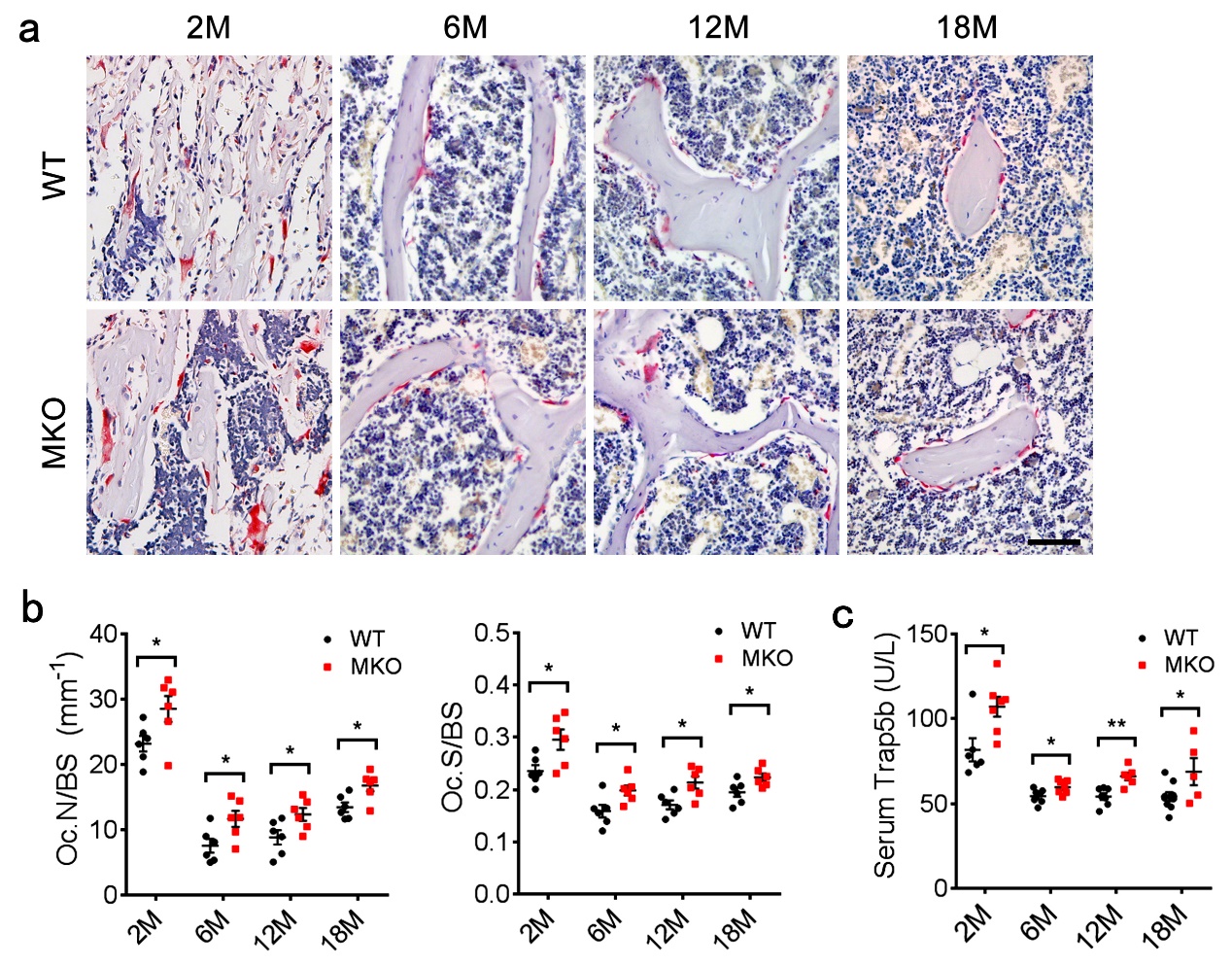


**Fig. S5. Depletion of CUL4B in MSCs enhances bone resorption in skeletal aging.** (**a**) Representative TRAP staining of osteoclasts surrounding trabecular bones of femurs from 2-, 6-, 12- and 18-month-old WT and MKO mice. Scale bar, 40 μm. (**b**) Analysis of osteoclast number (Oc.N/BS) and osteoclast surface (Oc.S/BS) surrounding trabecular bones of femurs from 2-, 6-, 12- and 18-month-old WT and MKO mice (*n*=6). Error bars represent standard errors. **p*<0.05. (**c**) ELISA of Trap5b in serum from 2- (*n*=6;7), 6- (*n*=7;7), 12- (*n*=7;6) and 18-month-old (*n*=9;5) WT and MKO mice. Error bars represent standard errors. **p*<0.05; ***p*<0.01.


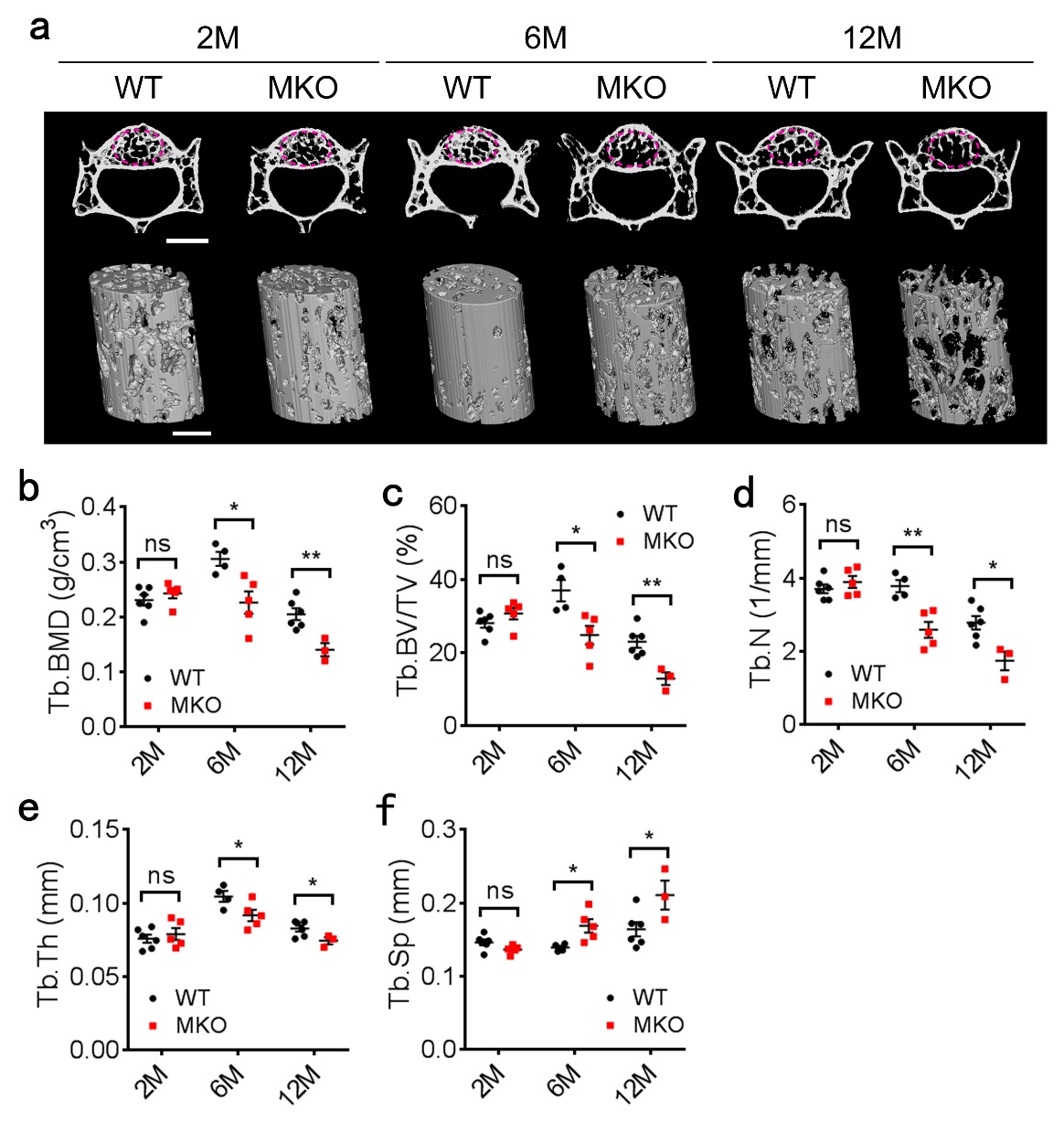


**Fig. S6. Micro-CT analysis of vertebrae.** (**a**) Representative micro-CT three-dimensional reconstruction images of lumber 4 (L4) vertebrae from 2-, 6- and 12-month-old MKO and littermate control mice. Scale bar, 1mm (upper) or 500μm (lower). The magenta circles indicate the region of interest. (**b-f**) Quantitative measurements of the trabecular bone mineral density (BMD) (**b**), bone volume/total volume (BV/TV) (**c**), trabecular number (Tb.N) (**d**), trabecular thickness (Tb.Th) (**e**) and trabecular spacing (Tb.Sp) (**f**) of lumber 4 (L4) vertebrae from 2- (*n*=6;5), 6- (*n*=4;5) and 12-month-old (*n*=6;3) WT and MKO mice by micro-CT. Error bars represent standard errors. **p*<0.05; ***p*<0.01; ns, no significance.


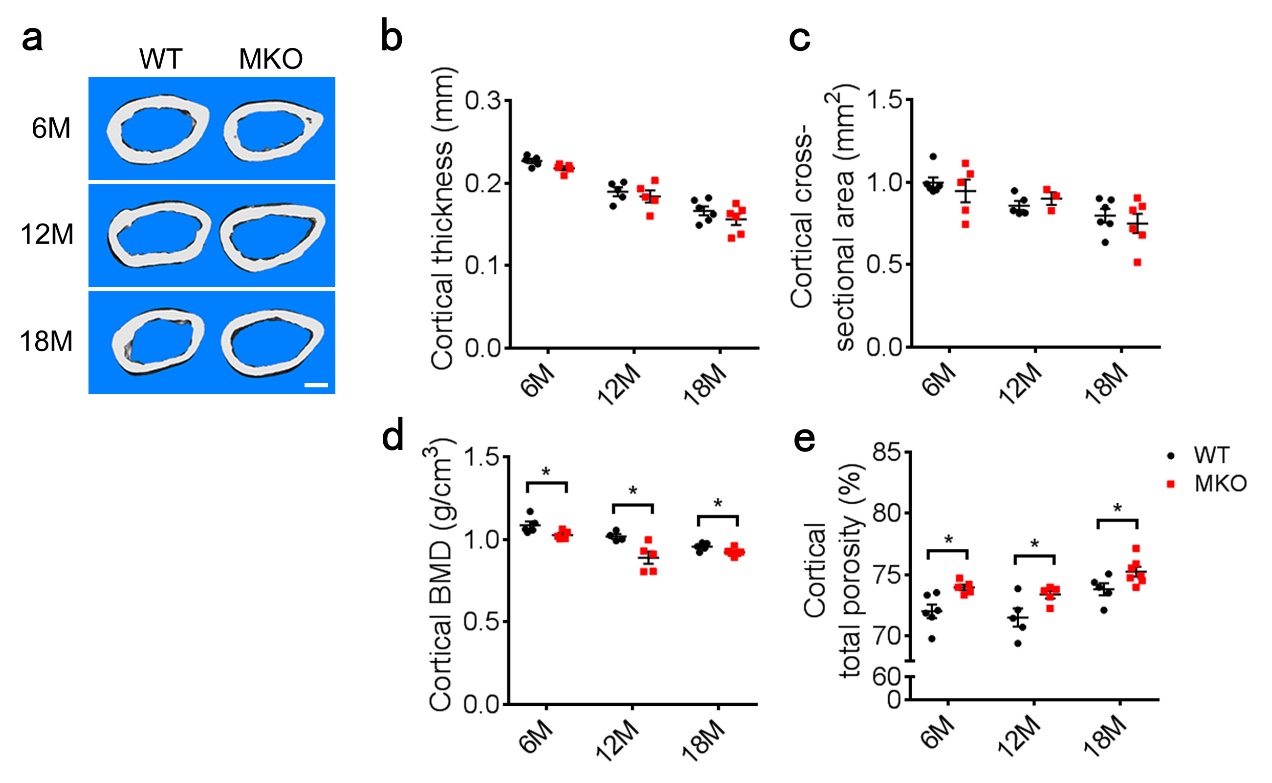


**Fig. S7. Micro-CT analysis of cortical bone in 6, 12, and 18 months.** (**a**) Representative micro-CT three-dimensional reconstruction images of cortical bone of femurs from 6-, 12- and 18-month-old WT and MKO mouse. Scale bar, 0.5 mm. (**b**) Quantification of cortical thickness of femurs from 6- (*n*=5;5), 12- (*n*=5;5) and 18-month-old (*n*=6;6) WT and MKO mouse. (**c**) Quantification of cortical cross-sectional area of femurs from 6- (n=6;5), 12- (n=5;3) and 18-month-old (n=6;6) WT and MKO mouse. (**d**) Quantitative measurements of the cortical bone mineral density (BMD) of femurs from 6- (*n*=5;5), 12- (*n*=4;5) and 18-month-old (*n*=5;6) WT and MKO mice by micro-CT. (**e**) Quantitative measurements of the cortical total porosity of femurs from 6- (n=6;5), 12- (n=5;5) and 18-month-old (n=5;7) WT and MKO mouse by micro-CT. Error bars represent standard errors. **p*<0.05.


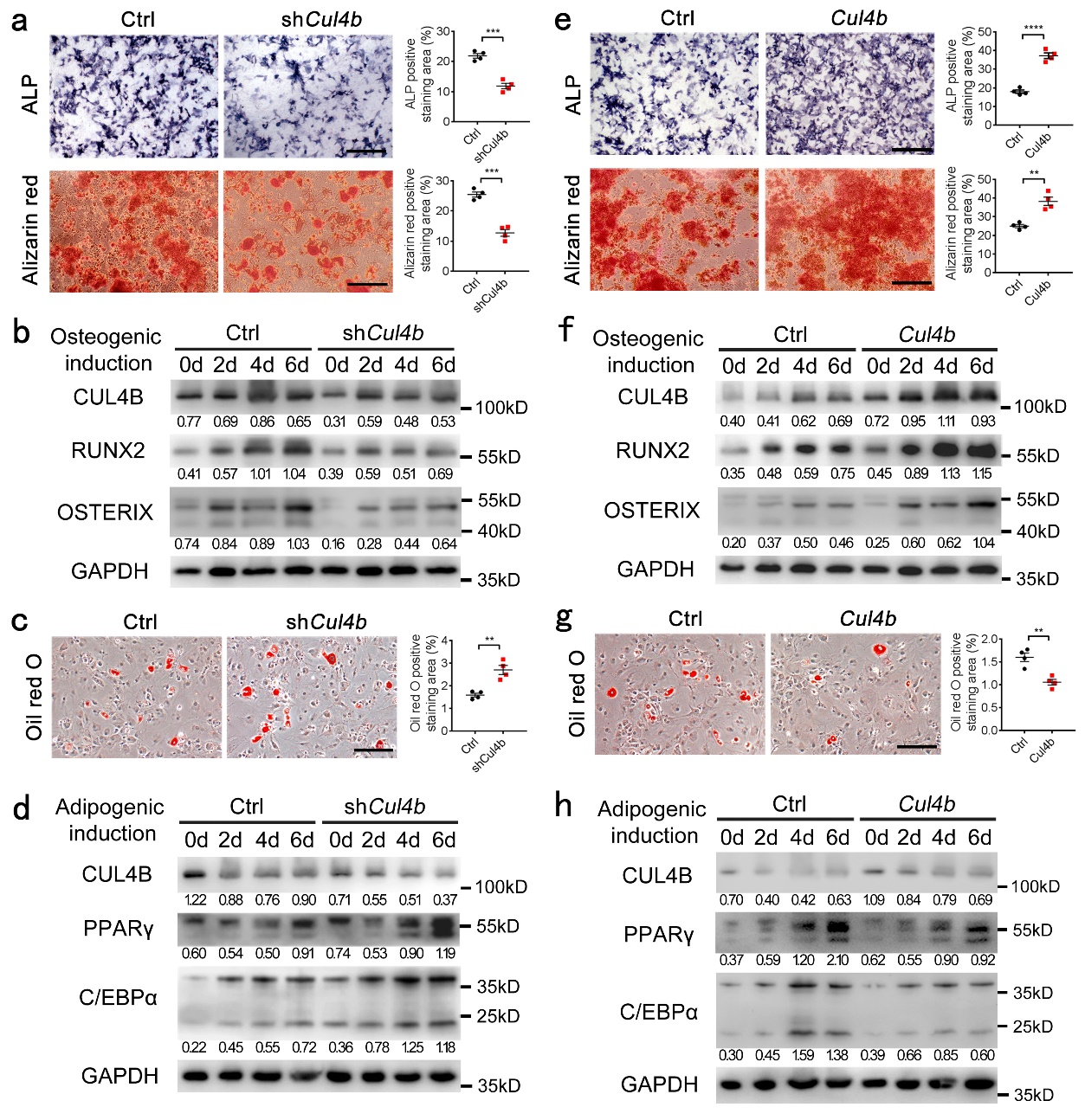


**Fig. S8. CUL4B regulates osteogenic and adipogenic differentiation of ST2 cells.** (**a**) Osteogenic differentiation of *Cul4b* knockdown and control ST2 cells (*n*=5) was determined by ALP staining (upper panels) and alizarin red staining (lower panels). Scale bar, 200 μm. (**b**) The protein levels of key osteogenic regulators RUNX2 and OSTERIX in *Cul4b* knockdown and control ST2 cells during osteogenic differentiation were detected by Western blotting. (**c**) Adipogenic differentiation of *Cul4b* knockdown and control ST2 cells (*n*=5) was determined by oil red O staining. Scale bar, 60 μm. (**d**) The protein levels of key adipogenic regulators PPARγ and C/EBPα in *Cul4b* knockdown and control ST2 cells during adipogenic differentiation were detected by Western blotting. (**e**) Osteogenic differentiation of CUL4B-overexpressing and control ST2 (*n*=5) cells was determined by ALP staining (upper panels) and alizarin red staining (lower panels). Scale bar, 200 μm. (**f**) The protein levels of key osteogenic regulators RUNX2 and OSTERIX in CUL4B-overexpressing and control ST2 cells during osteogenic differentiation were detected by Western blotting. (**g**) Adipogenic differentiation of CUL4B-overexpressing and control ST2 cells (*n*=5) was determined by oil red O staining. Scale bar, 60 μm. (**h**) The protein levels of key adipogenic regulators PPARγ and C/EBPα in CUL4B-overexpressing and control ST2 cells during adipogenic differentiation were detected by Western blotting. Error bars represent standard errors. ***p*<0.01; ****p*<0.001; *****p*<0.0001.


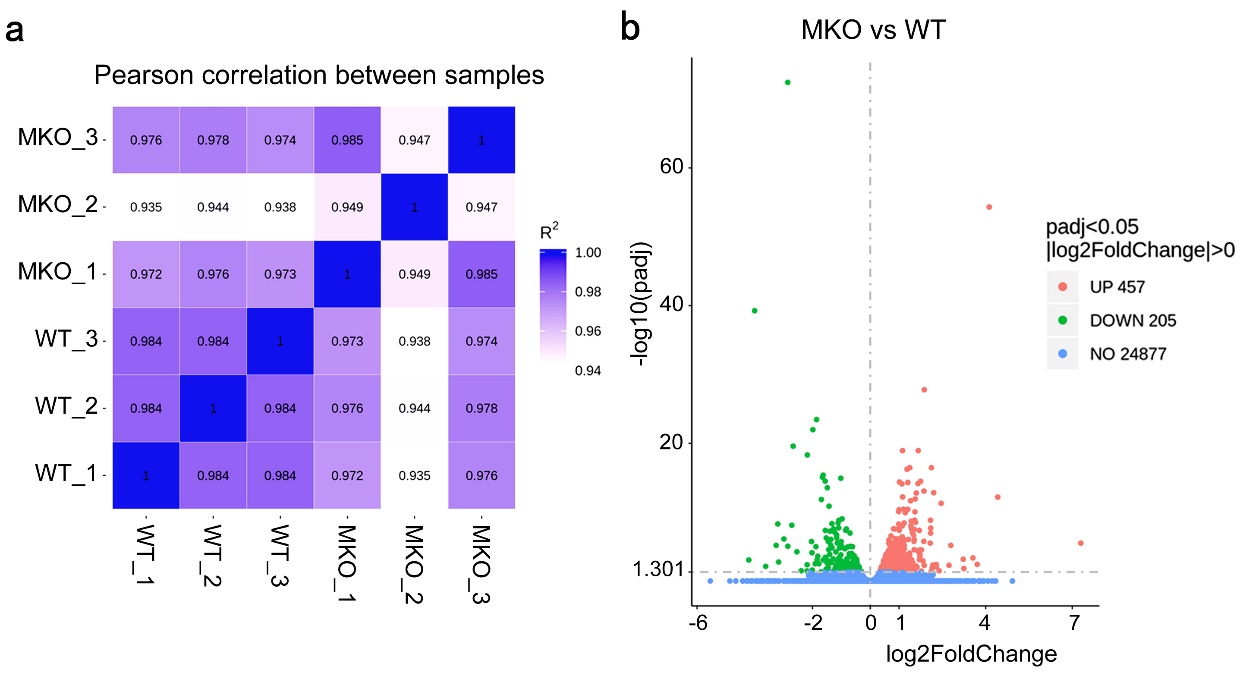


**Fig. S9. Data analysis of RNA-seq.** (**a**) Heat map of pearson correlation coefficients of all samples. (**b**) Volcano map of all differentially expressed genes (DEGs).

**Table S1. The sequences of primers for quantitative real-time RT-PCR.**

| Primers | Sequences |
| --- | --- |
| *Cul4b* | F：5’-TATTAGTTGGCAAGAGTGCAT-3’ |
|  | R：5’-CCAGTAACCCATTGTCAGGAT-3’ |
| *Runx2* | F：5’-ATGCTTCATTCGCCTCACAAA-3’ |
|  | R：5’-GCACTCACTGACTCGGTTGG-3’ |
| *Osterix* | F：5’-ATGGCGTCCTCTCTGCTTG-3’ |
|  | R：5’-TGAAAGGTCAGCGTATGGCTT-3’ |
| *Alpl* | F：5’-CCAACTCTTTTGTGCCAGAGA-3’ |
|  | R：5’-GGCTACATTGGTGTTGAGCTTTT-3’ |
| *Ibsp* | F：5’-CAGGGAGGCAGTGACTCTTC-3’ |
|  | R：5’-AGTGTGGAAAGTGTGGCGTT-3’ |
| *Bglap* | F：5’-AAGCAGGAGGGCAATAAGGT-3’ |
|  | R：5’-ATGCGTTTGTAGGCGGTCTT-3’ |
| *Pparg* | F：5’-TCGCTGATGCACTGCCTATG-3’ |
|  | R：5’-GAGAGGTCCACAGAGCTGATT-3’ |
| *Cebpa* | F：5’-CAAGAACAGCAACGAGTACCG-3’ |
|  | R：5’-GTCACTGGTCAACTCCAGCAC-3’ |
| *Fabp4* | F：5’-AAGGTGAAGAGCATCATAACCCT-3’ |
|  | R：5’-TCACGCCTTTCATAACACATTCC-3’ |
| *Adipoq* | F：5’-CGTCACTGTTCCCAATGT-3’ |
|  | R：5’-ACCGTGATGTGGTAAGAG-3’ |
| *Plin1* | F：5’-CCTGTGGTGAGCGGGACC-3’ |
|  | R：5’-CCTGTGGTGAGCGGGACC-3’ |
| *Sox5* | F：5’-TTTTCCCAACAAGCCTCACTC-3’ |
|  | R：5’-CCAGTAACCCATTGTCAGGAT-3’ |
| *Sall1* | F：5’-AGTCGCCCCACTAAGAGCA-3’ |
|  | R：5’-GGGAGGCTGGACTTTCATTGA-3’ |
| *Col2a1* | F：5’-TGAAGACCCAGACTGCCTCAA-3’ |
|  | R：5’-AGCCGCGAAGTTCTTTTCTCC-3’ |
| *Acan* | F：5’-CCTGCTACTTCATCGACCCC-3’ |
|  | R：5’-AGATGCTGTTGACTCGAACCT-3’ |
| *Ucma* | F：5’-GAATCTGATGCCTCCAATTTCCT-3’ |
|  | R：5’-TCGTTCCTTTGCTCCTCGTAATA-3’ |
| *Mmp13* | F：5’-CTTCTTCTTGTTGAGCTGGACTC-3’ |
|  | R：5’-CTGTGGAGGTCACTGTAGACT-3’ |
| *Fzd9* | F：5’-GTGCCAAGCGATGGAGATCC-3’ |
|  | R：5’-GCGTAGAGCGAGCAGAAGAA-3’ |
| *Cnmd* | F：5’-CCTGAGGACGTTGAGTTTTGC-3’ |
|  | R：5’-CAGCTCCTACCTTGAGCAGC-3’ |
| *Tgfb2* | F：5’-TCGACATGGATCAGTTTATGCG-3’ |
|  | R：5’-CCCTGGTACTGTTGTAGATGGA-3’ |
| *Ptprq* | F：5’-ATTTCTGCCACAACCTACAGC-3’ |
|  | R：5’-GGAGGGGTATTCCATGAAAGGAG-3’ |
| *Dlx1* | F：5’-ATGCCAGAAAGTCTCAACAGC-3’ |
|  | R：5’-AACAGTGCATGGAGTAGTGCC-3’ |
| *Medag* | F：5’-GGCCTTGTGCGCCTAGAAG-3’ |
|  | R：5’-TGCTCAGTATCGTTTCCCTGTA-3’ |
| *Klf4* | F：5’-GTGCCCCGACTAACCGTTG-3’ |
|  | R：5’-GTCGTTGAACTCCTCGGTCT-3’ |
| *Cebpd* | F：5’- CGACTTCAGCGCCTACATTGA-3’ |
|  | R：5’- CTAGCGACAGACCCCACAC-3’ |
| *Hes1* | F：5’-CCAGCCAGTGTCAACACGA-3’ |
|  | R：5’-AATGCCGGGAGCTATCTTTCT-3’ |
| *Sfrp1* | F：5’-CAACGTGGGCTACAAGAAGAT-3’ |
|  | R：5’-GGCCAGTAGAAGCCGAAGAAC-3’ |
| *Bmper* | F：5’-GCCTGGGATTACCTGCTGC-3’ |
|  | R：5’-ACACATTATGCAAGGGTTGTCTG-3’ |
| *Grem2* | F：5’-GGTAGCTGAAACACGGAAGAA-3’ |
|  | R：5’-TCTTGCACCAGTCACTCTTGA-3’ |
| *Pmepa1* | F：5’-TGGAGTTCGTGCAAATCGTG-3’ |
|  | R：5’-GCTGTGTCGGCTGATGAAG-3’ |
| *Ecm1* | F：5’-GGGACCGTATCCAGAGCAG-3’ |
|  | R：5’-GCTGGTCTGAAGCCTTGAAG-3’ |
| *Chrdl2* | F：5’-TTTGCTGGGACTCGTGATGTT-3’ |
|  | R：5’-GTGGTTCCAAGTAGGGGTGC-3’ |
| *Vsir* | F：5’-GGAACCCTGCTCCTTGCTATT-3’ |
|  | R：5’-TTGTAGATGGTCACATCGTGC-3’ |
| *Gapdh* | F：5’-AGGTCGGTGTGAACGGATTTG-3’ |
|  | R：5’-TGTAGACCATGTAGTTGAGGTCA-3’ |

**Table S2. The sequences of primers for quantitative ChIP.**

| Primers | Sequences |
| --- | --- |
| *Klf4*-ChIP-1 | F：5’-CCAGGTAAGGACATCAGCC-3’ |
| (-10248 ~ -10100) | R：5’-TGACAATAAATAGCCAAGGAAAC-3’ |
| *Klf4*-ChIP-2 | F：5’-GCCTCTGATTCTGTGGGTGC-3’ |
| (-9039 ~ -8870) | R：5’-GTCTCCCAGTTATTGTCCTCCAGTA-3’ |
| *Klf4*-ChIP-3 | F：5’-TGGACTGCCCTCCTTGAAT-3’ |
| (-8255 ~ -8083) | R：5’-TTTTGCACGTCTCGTAGTGTATT-3’ |
| *Klf4*-ChIP-4 | F：5’-GGGAGGAGAAAGAGGGAGAT-3’ |
| (-7474 ~ -7362) | R：5’-GGAGGTGGAGGCTGGAAGA-3’ |
| *Klf4*-ChIP-5 | F：5’-GGCTCTTCACCCACTTTGTC-3’ |
| (-6067 ~ -5943) | R：5’-TTTATTCCTCTTACCCATTCATTT-3’ |
| *Klf4*-ChIP-6 | F：5’-TTCAGAAATACTCAGGGCACAG-3’ |
| (-4452 ~ -4431) | R：5’-CAGGCACAATAAACTGGAGATAG-3’ |
| *Klf4*-ChIP-7 | F：5’-AGGTGCTTCTGCTTCGGC-3’ |
| (-4065 ~ -4048) | R：5’-ATGCGTTCCTCCCACTTGA-3’ |
| *Klf4*-ChIP-8 | F：5’-CAATCGCAAGAAGAACCCG-3’ |
| (-2843 ~ -2825) | R：5’-ACCATCTAACCAGTAACCACCAC-3’ |
| *Klf4*-ChIP-9 | F：5’-ACCTAAGTTGATGGGAGCGAGT-3’ |
| (-1562 ~ -1541) | R：5’-GGAAGGAACAAGGAAGGCG-3’ |
| *Klf4*-ChIP-10 | F：5’-TCTGCCTTGCTGATTGTCTATTT-3’ |
| (-243 ~ -221) | R：5’-CCCCAGATTGCCCGAGAT-3’ |
| *Klf4*-ChIP-11 | F：5’-CTGCTCCCGTCCTTCTCCA-3’ |
| (162 ~ 346) | R：5’-AGGGCAATCCTTGTCCTAACC-3’ |
| *Cebpd*-ChIP-1 | F：5’-GGGAAGAGGAAGATAGGGAGAG-3’ |
| (-8726 ~ -8872) | R：5’-CGCTGAGACTTGCTGCTGAATA-3’ |
| *Cebpd*-ChIP-2 | F：5’-ATCCCATCTACTCCTCCCATCTT-3’ |
| (-7581 ~ -7708) | R：5’-TGTTTTGCCTTGTCCGACTTTA-3’ |
| *Cebpd*-ChIP-3 | F：5’-AAGCAGCAGTGAGCATAGTTGAG-3’ |
| (-5645 ~ -5838) | R：5’-TAGCCAGGATGTAGAGTAAAGGGA-3’ |
| *Cebpd*-ChIP-4 | F：5’-ATGTGTTTTTAGATTCAGTGTTCAAGC-3’ |
| (-4920 ~ -5031) | R：5’-AGCATTTATTCAACATCTCCCAAC-3’ |
| *Cebpd*-ChIP-5 | F：5’-AGTTAGGGTTTTGATTATTATGTCC-3’ |
| (-4102 ~ -4283) | R：5’-GAATAGAGGAGAGAAAAGAAACCCA-3’ |
| *Cebpd*-ChIP-6 | F：5’-CAGGAGGTATGGGTAGGGGAG-3’ |
| (-2879 ~ -3012) | R：5’-CAGACCAGGGAGTCAGGTAAGC-3’ |
| *Cebpd*-ChIP-7 | F：5’-CCGCCATTTATTACAACGTTC-3’ |
| (-2243 ~ -2336) | R：5’-AAGAAGTGAAGGCAGGAGGAT-3’ |
| *Cebpd*-ChIP-8 | F：5’-GTCCTTGAGATTGGCACAGTG-3’ |
| (-1483 ~ -1626) | R：5’-CAGAAATGGAGAAAGGCAACA-3’ |
| *Cebpd*-ChIP-9 | F：5’-GGAGGGATATGAGGGACACA-3’ |
| (-815 ~ -928) | R：5’-TGATAGGCTAGGTGGGTAGTTGA-3’ |
| *Cebpd*-ChIP-10 | F：5’-TTTAATAACCAAGAAGAAATGCCAG-3’ |
| (-452 ~ -613) | R：5’-GAGGTTAGGTTCAGCAACAGAGC-3’ |
| *Cebpd*-ChIP-11 | F：5’-AGTTCTGGTCTCGTGGCGG-3’ |
| (-148 ~ -321) | R：5’-CCTTCCTGTTTGTGCGGTTT-3’ |
| *Cebpd*-ChIP-12 | F：5’-CTACGAGCCAGGCAGGGTG-3’ |
| (72 ~ 258) | R：5’-GAAGAGGTCGGCGAAGAGTT-3’ |
